# High-resolution genetic mapping of allelic variants associated with cell wall chemistry in *Populus*

**DOI:** 10.1186/s12864-015-1215-z

**Published:** 2015-01-23

**Authors:** Wellington Muchero, Jianjun Guo, Stephen P DiFazio, Jin-Gui Chen, Priya Ranjan, Gancho T Slavov, Lee E Gunter, Sara Jawdy, Anthony C Bryan, Robert Sykes, Angela Ziebell, Jaroslav Klápště, Ilga Porth, Oleksandr Skyba, Faride Unda, Yousry A El-Kassaby, Carl J Douglas, Shawn D Mansfield, Joel Martin, Wendy Schackwitz, Luke M Evans, Olaf Czarnecki, Gerald A Tuskan

**Affiliations:** BioEnergy Science Center, Oak Ridge National Laboratory, Oak Ridge, TN 37831 USA; Department of Biology, West Virginia University, Morgantown, WV 26506 USA; Institute of Biological, Environmental and Rural Sciences, Aberystwyth University, Aberystwyth, SY23 3EB UK; Bioscience Center, National Renewable Energy Laboratory, 15013 Denver West Parkway, Golden, CO 80401 USA; Department of Forest and Conservation Sciences, Faculty of Forestry, University of British Columbia, Forest Sciences Centre, 2424 Main Mall, Vancouver, BC V6T 1Z4 Canada; Department of Wood Science, Faculty of Forestry, University of British Columbia, Forest Sciences Centre, 2424 Main Mall, Vancouver, BC V6T 1Z4 Canada; Department of Botany, University of British Columbia, Vancouver, BC V6T 1Z4 Canada; U.S. Department of Energy Joint Genome Institute, Walnut Creek, CA 94598 USA; Current address: Department of Plant Biology, Carnegie Institute for Science, Stanford, CA 94305 USA; Department of Genetics and Physiology of Forest Trees, Faculty of Forestry and Wood Sciences, Czech University of Life Sciences in Prague, Kamýcká 129, 165 21 Praha 6, Czech Republic

**Keywords:** QTL cloning, Association genetics, Cell wall recalcitrance, Lignin, Cellulose, Hemicellulose

## Abstract

**Background:**

QTL cloning for the discovery of genes underlying polygenic traits has historically been cumbersome in long-lived perennial plants like *Populus*. Linkage disequilibrium-based association mapping has been proposed as a cloning tool, and recent advances in high-throughput genotyping and whole-genome resequencing enable marker saturation to levels sufficient for association mapping with no *a priori* candidate gene selection. Here, multiyear and multienvironment evaluation of cell wall phenotypes was conducted in an interspecific *P. trichocarpa* x *P. deltoides* pseudo-backcross mapping pedigree and two partially overlapping populations of unrelated *P. trichocarpa* genotypes using pyrolysis molecular beam mass spectrometry, saccharification, and/ or traditional wet chemistry. QTL mapping was conducted using a high-density genetic map with 3,568 SNP markers. As a fine-mapping approach, chromosome-wide association mapping targeting a QTL hot-spot on linkage group XIV was performed in the two *P. trichocarpa* populations. Both populations were genotyped using the 34 K *Populus* Infinium SNP array and whole-genome resequencing of one of the populations facilitated marker-saturation of candidate intervals for gene identification.

**Results:**

Five QTLs ranging in size from 0.6 to 1.8 Mb were mapped on linkage group XIV for lignin content, syringyl to guaiacyl (S/G) ratio, 5- and 6-carbon sugars using the mapping pedigree. Six candidate loci exhibiting significant associations with phenotypes were identified within QTL intervals. These associations were reproducible across multiple environments, two independent genotyping platforms, and different plant growth stages. cDNA sequencing for allelic variants of three of the six loci identified polymorphisms leading to variable length poly glutamine (PolyQ) stretch in a transcription factor annotated as an ANGUSTIFOLIA C-terminus Binding Protein (CtBP) and premature stop codons in a KANADI transcription factor as well as a protein kinase. Results from protoplast transient expression assays suggested that each of the polymorphisms conferred allelic differences in the activation of cellulose, hemicelluloses, and lignin pathway marker genes.

**Conclusion:**

This study illustrates the utility of complementary QTL and association mapping as tools for gene discovery with no *a priori* candidate gene selection. This proof of concept in a perennial organism opens up opportunities for discovery of novel genetic determinants of economically important but complex traits in plants.

**Electronic supplementary material:**

The online version of this article (doi:10.1186/s12864-015-1215-z) contains supplementary material, which is available to authorized users.

## Background

The genus *Populus* represents an economically and ecologically important set of species whose uses include pulp and paper, carbon sequestration, phytoremediation, and, more recently, feedstock for the lignocellulosic biofuels industry [1,2]. However, a barrier to widespread adoption of *Populus* and other plants as a biofuels feedstock is the inherent recalcitrance of cell walls to enzymatic digestion and microbial deconstruction into simple sugars. This recalcitrance leads to increased processing costs for conversion of biomass into liquid fuels [3]. Genetic manipulation of biomass to enhance sugar release from the harvested aerial portion of plants has been a key approach in efforts to establish an economically sustainable lignocellulosic biofuels industry [4,5].

Quantitative trait locus (QTL) studies have been successfully used in *Populus* to identify genomic regions associated with cell wall recalcitrance phenotypes including lignin content, syringyl-to-guaiacyl (S/G) ratio, and 5- and 6-carbon sugar content [6,7]. However, QTL mapping approaches do not have sufficient mapping resolution to identify the causal genes due to extensive linkage and the large concomitant genomic intervals that typically encompass dozens to hundreds of genes. Regardless of this limitation, QTL analysis provides a useful approach to mitigate false positives in genetic mapping studies. As a complementary approach, linkage disequilibrium (LD)-based association mapping has been proposed as a fine mapping approach with potential for single-gene resolution [8].

Recent studies in *Populus* have demonstrated the feasibility of association mapping by *a priori* targeting genes with known function in cell wall biosynthesis [9-12]. These studies have characterized the extent of LD over physical distance and reported r^2^ dropping to 0.1 within 200 to 700 bp. However, Guerra et al. observed distances up to 2.5 kb for a single candidate gene [10]. The latter estimate was in close agreement with findings reported by Slavov et al. in a population of unrelated *P. trichocarpa* genotypes, where r^2^ dropped below 0.2 within 3–6 kb on a whole-genome scale, as well as in the vicinity of coding regions [13]. Given that the average gene size for *P. trichocarpa* is approximately 5 kb, these results suggest that fine-scale mapping using association approaches in *Populus* should be possible.

In this study, we sought to identify loci underlying cell wall recalcitrance phenotypes using a sequential combination of *P. trichocarpa* x *P. deltoides* pseudo-backcross-based QTL mapping and then association mapping among two partially overlapping populations of unrelated *P. trichocarpa* genotypes to assess co-location of significant SNP trait associations within QTL intervals. We also report on the reproducibility of associations across alternate phenotyping and genotyping platforms, as well as across multiple sampling environments and varying plant ages. Finally, using the *Populus* protoplast transient expression assay, we demonstrate the effects of allelic variants of candidate genes on reporter gene expression for cellulose, hemicellulose, and lignin biosynthetic pathways.

## Results

### Phenotyping for cell wall traits

There was significant phenotypic variation in all populations for cell wall traits analyzed in this study. In the pseudo-backcross pedigree, which was characterized using pyrolysis molecular beam mass spectrometry (pyMBMS), phenotypic values for each trait were significantly correlated within and between age 2 and age 4 plant materials (Additional file 1A). Additionally, we observed correlations between different phenotypes which were generally higher within the same environment. For example, lignin and S/G ratio were significantly correlated in age 2 plants (r = 0.37, p ≤ 0.00001) and age 3 plants (r = 0.36, p ≤ 0.00001), and 5- and 6-carbon sugars were negatively correlated with lignin content in the age 2 plants (r = −0.65, p ≤ 0.00001 and r = −0.77, p ≤ 0.00001, respectively).

The BESC association mapping population was characterized using pyMBMS and saccharification assays using wood samples collected from the native, Corvallis, and Clatskanie environments; the Surrey population was characterized using traditional wet chemistry using samples collected from the Surrey field site. Phenotypic correlations were generally higher within the same environment with varying levels of significance across different environments (Additional files 1B and C). The S/G ratio exhibited the highest correlation across different environments, achieving r = 0.43, p ≤ 0.00001 (n = 258) between the Corvallis and Clatskanie common gardens and r = 0.31, p ≤ 0.00001 (n = 795) between the Clatskanie and native environments. Similarly, the S/G ratio had the highest correlation between different phenotyping platforms, reaching r = 0.61, p ≤ 0.00001 (n = 146) between the pyMBMS-characterized native and the wet chemistry-characterized Surrey environments (Additional file 1D). Glucose release was negatively correlated with lignin content in both native and Clatskanie environments as well as between the native environments and the Surrey populations phenotyped using different platforms (Additional file 1D). However, the marginal to low levels of correlation suggest significant environmental and developmental effects on trait expression. Details for phenotyping characteristics are provided in Additional file 2.

### SNP genotyping in pseudo-backcross pedigree and genetic map construction

We incorporated 3,568 of the 3,751 segregating SNP markers into 19 linkage groups (LGs) corresponding to the 19 *Populus* chromosomes. The map was 3,053.9 cM in length, with the longest linkage group being 3791.2 cM for LG I and the shortest being 98.7 cM for LG XIX. The number of markers in a single linkage group ranged from 93 for LG XII to 458 for LG I. The average marker distance was 0.8 cM, and the map covered 90% of the *P. trichocarpa* reference genome. The target LG XIV had 180 SNP markers, with a median marker distance of 0.5 cM and an average of 0.8 cM. The largest marker distance was 5.8 cM, and only 15 intervals had distances greater than 2.0 cM.

### SNP genotyping in *P. trichocarpa* populations

Array performance data for the 34 K Illumina Infinium® SNP array are described in detail by Geraldes et al. [14], and SNP genotyping results for the Surrey population are described by Porth et al. [12,15]. For the BESC population, of the 34 K SNPs, 27,940 had <10% missing data, with MAF across all loci ranging from 0.044 to 0.500. On the target chromosome XIV, 1,439 SNPs met the minimum criteria (i.e., <10% missing data and MAF ≥0.05) for use in association mapping.

### QTL mapping

**Figure 1:**
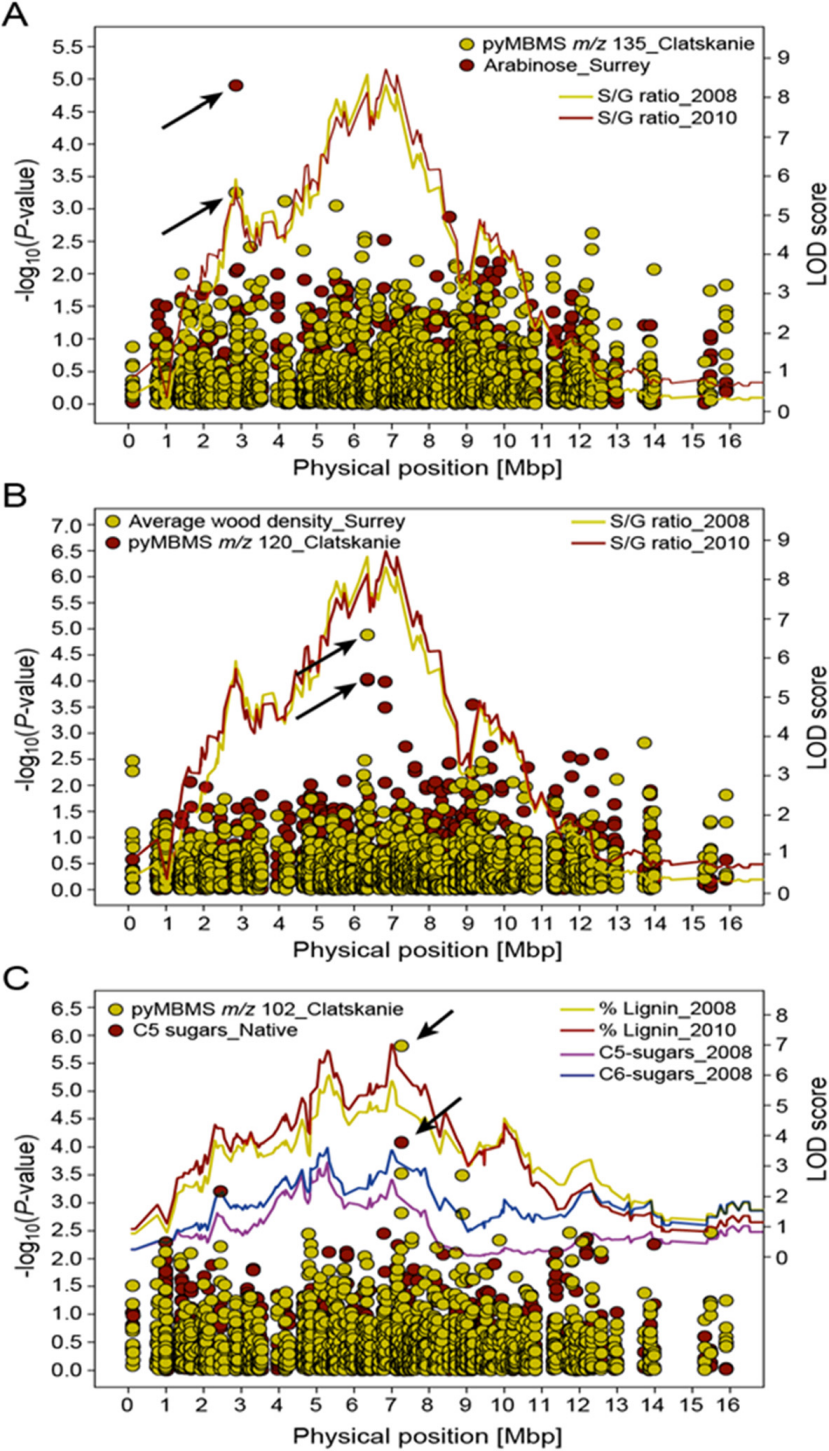
**LOD score profiles (solid lines) showing QTLs on** ***Populus*** **chromosome XIV for lignin content, S/G ratio, 5-carbon (C5) and 6-carbon (C6) sugars and co-location of QTL peaks with SNP-trait associations (closed circles) for the (A) amino acid transporter, (B) copper transport ATOX1-like, and (C) Ca** ^**2+**^ **transporting ATPase genes.**

Five-hundred-and-fifteen genotypes from the pseudo-backcross population, with both phenotypic and genotypic data, were used in QTL mapping. A broad QTL hotspot for lignin content, S/G ratio, and 5- and 6-carbon sugars was identified on linkage group XIV corresponding to chromosome 14 of the *Populus* genome (Figure 1). Using a drop in LOD score of 1 between peaks to distinguish neighboring QTLs, we identified five putative QTLs each for S/G ratio and lignin content and three for 5- and 6-carbon sugars (Additional file 3). All QTLs exceeded the genome-wise LOD significance thresholds for each phenotype in each experiment with percentage phenotypic variance explained (% PVE) ranging from 1.9 to 7.5%. QTL profiles were reproducible between phenotypic data sets collected in two different years on 2- and 4-year-old progeny, respectively (Figure 1). Individual SNPs co-locating with QTL peaks were highly consistent between age 2 and age 4 plant materials. Lignin content and 5- and 6-carbon sugar contents had the most robust co-location of QTL peaks. In each case, the same SNP markers had the highest LOD scores for each phenotype in each experiment/year (Additional file 3). The two largest QTLs occurred adjacent to each other and were both associated with S/G ratio, achieving LOD scores above 8 in each case. SNP markers scaffold_14_7068969 and scaffold_14_7559196 were associated with the QTL peaks in both the 2008 and 2010 phenotypic datasets, both exceeding 7% PVE. QTL intervals ranged from 0.559 to 1.766 Mb (Additional file 3).

### Population structure within the association population

After excluding genotypes exhibiting evidence of clonality and high levels of relatedness for the BESC population, we analyzed a set of 886 genotypes for population structure. There was a substantial increase in probability In P(D) as a function of number of subpopulations from *K* = 1 up to *K* = 6. The smallest differences among In P(D) values were observed from *K* = 7 up to *K* = 10, after which the values exhibited substantial decrease between *K* = 11 and *K* = 15. We selected *K* = 10, which had the highest ln P(D), as the number of subpopulations in the Q matrix generated as a covariate in association analysis. Attributes of the Surrey population are described by Porth et al. [12].

### Association mapping

From the *Populus* 34 K Infinium array-based association mapping effort, we identified seven SNPs within six candidate genes which exceeded the chromosome-wide –log_10_(*P*) = 4.46 [p = 3.47E^−05^ (*p* ≤ 0.05)] Bonferroni-adjusted significance threshold (Additional file 4). Each of the six genes also harbored SNPs exhibiting significance when correction for false positives was conducted at the QTL interval level. Altogether, twelve SNPs from six candidate genes were ranked first in 14 unique marker-trait associations across the four sampling environments (Additional file 4). Reanalysis of candidate gene intervals saturated using whole-genome resequencing data identified 21 SNPs from five of the six intervals with significant trait associations (Table 1 and Additional file 5). SNPs from the resequencing data set with the lowest *p*-values mapped within 10.0 kb or less across multiple environments for five of the six intervals identified using the Infinium array data (Table 1). For the single remaining interval which encompassed a 17.9 kb-candidate gene, SNPs mapped within 1.5 kb across all three environments for the Infinium array and within 30.7 kb across two environments for resequencing-based associations (Table 1). Significant associations were detected within QTL intervals for all phenotypes noted above (i.e., S/G ratio, lignin content, and 5- and 6-carbon sugars).

**Table 1:** **SNP trait associations with the highest significance across different environments for six candidate intervals**

**Nearest gene**	**Environment**	**Infinium array**			**Re-sequencing**		
		**SNP marker**	***p*** **-value**	**Trait**	**SNP marker**	***p*** **-value**	**Trait**
Potri.014G036500	Clatskanie	scaffold_14_2980220	1.27E^−05^	pyMBMS *m/z* 135	scaffold_14_2986800	6.54E^−05^	Glucose release
(Amino acid transporter)	Corvallis	-	-	-	scaffold_14_2983445	1.14E^−07^	6-carbon sugars
	Surrey	scaffold_14_2977633	5.63E^−04^	Arabinose	-	-	-
Potri.014G037200	Clatskanie	scaffold_14_3028570	8.55E^−04^	pyMBMS *m/z* 125	scaffold_14_3025294	8.89E^−05^	Glucose release
(KANADI TF)	Corvallis	-	-	-	scaffold_14_3035343	8.64E^−05^	Glucose release
	Native	scaffold_14_3028120	6.57E^−06^	Glucose/xylose release	scaffold_14_3029359	4.43E^-04*^	Glucose/xylose release
Potri.014G089400	Corvallis	scaffold_14_7043301	1.06E^−05^	Xylose release	scaffold_14_7041563	4.63E^-04*^	5-carbon sugars
(ANGUSTIFOLIA TF)	Native	scaffold_14_7044284	6.84E^−04^	Glucose/xylose release	scaffold_14_7044259	5.36E^-04*^	6-carbon sugars
Potri.014G089700	Clatskanie	scaffold_14_7055338	1.32E^−05^	pyMBMS *m/z* 120	scaffold_14_7053121	1.96E^−05^	6-carbon sugars
(Copper transporter)	Corvallis	-	-	-	scaffold_14_7062644	3.20E^-04*^	Xylose release
	Native	-	-	-	scaffold_14_7054885	3.36E^-04*^	S/G ratio
	Surrey	scaffold_14_7053760	9.18E^−05^	Average wood density	-	-	-
Potri.014G101900	Clatskanie	scaffold_14_7971054	8.31E^−05^	pyMBMS *m/z* 102	-	-	-
(Ca2 + −transporting ATPase)	Corvallis	-	-	-	scaffold_14_7970015	9.03E^−06^	Xylose release
	Native	scaffold_14_7969314	1.55E^−06^	5-carbon sugars	scaffold_14_7966546	5.61E^−05^	5-carbon sugars
Potri.014G142700	Clatskanie	scaffold_14_10865898	3.00E^−05^	pyMBMS *m/z* 235	scaffold_14_10886410	2.42E^-04*^	S/G ratio
(Protein kinase)	Corvallis	-	-	-	scaffold_14_10855744	5.49E^−07^	Xylose release
	Native	scaffold_14_10867394	5.59E^−04^	Glucose release	-	-	-
	Surrey	scaffold_14_10865898	7.99E^−05^	Syringyl monomers	-	-	-

^*^Suggestive associations not significant at the Bonferroni-adjusted *p*-value.

### Candidate genes

An amino acid transporter, Potri.014G036500, harbored a SNP, scaffold_14_2979511, which had a LOD score of 5.72 and was at the peak position of the QTL identified for S/G ratio in the pseudo-backcross population (Additional file 3, Figure 1A). In Clatskanie, the same gene harbored a SNP, scaffold_14_2980220, which was significantly associated with pyMBMS peak *m/z* = 135 (*p* = 1.27E^−05^). pyMBMS peak 135 is derived from 4-vinyl-guaiacol, a component of G lignin [16]. Four additional SNPs mapping within a 6.5 kb interval about this same gene had significant associations with MBMS-estimated 6-carbon sugars (*p* = 1.14E^−07^) and glucose/xylose release (*p* = 3.86E^−06^) in Corvallis, glucose release (*p* = 6.54E^−05^) in Clatskanie, and arabinose content (*p* = 5.63E^−04^) in Surrey (Additional file 4, Table 1).

A KANADI transcription factor, Potri.014G037200, harbored SNP markers scaffold_14_3027959 and scaffold_14_3028120 that showed significant associations with glucose/xylose (*p* = 6.57E^−06^) release and glucose release (*p* = 1.38E^−05^) based on a saccharification assay from wood samples collected from the parental trees in their native environments (Additional file 4, Table 1). SNPs found within a 10 kb region encompassing the transcription factor were also significantly associated with glucose release from wood samples collected in Clatskanie (*p* = 8.89E^−05^) and Corvallis (*p* = 8.64E^−05^) (Table 1).

An ANGUSTIFOLIA CtBP transcription factor, Potri.014G089400, harbored SNPs from the Infinium array that were significantly associated with xylose release (*p* = 1.06E^−05^) at the chromosome-wise threshold from wood samples collected from Corvallis and glucose/xylose release (*p* = 6.84E^−04^) at the QTL-wise threshold from wood samples collected from their native environment. There were no significant associations when we reanalyzed the same interval using SNPs from the resequencing effort. However, three SNPs spanning a 2.7 kb region had suggestive associations with glucose/xylose release (*p* = 6.84E^−04^) and 5-carbon sugars (*p* = 4.63E^-04^) in the native environments and 6-carbon sugars (*p* = 5.36E^−04^) in Corvallis.

A copper transport protein ATOX1-related gene, Potri.014G089700, was the nearest gene to the peak of a major QTL for S/G ratio which peaked on SNP scaffold_14_7068969 (Additional file 3). The gene itself contained two SNPs, scaffold_14_7053760 and scaffold_14_7054863, which ranked first out of 1,439 SNPs for two traits in the Clatskanie and Surrey sites (Additional file 4). Three significant and two suggestive associations were observed for SNPs within a 9.5 kb interval encompassing Potri.014G089700. These were associated with pyMBMS peak *mz* = 120 (*p* = 1.32E^−05^), 6-carbon sugars (*p* = 1.96E^−05^), average wood density (*p* = 9.18E^−05^), xylose release (*p* = 3.20E^−04^), and S/G ratio (*p* = 3.36E^−04^) across all four test environments (Additional file 4, Table 1).

Two SNPs mapping within a Ca^2+^ transporting ATPase, Potri.014G101900, had trait associations with *p*-values exceeding the chromosome-wise Bonferroni-adjusted significance threshold in two of the four environments. SNP marker scaffold_14_7969314 was associated with pyMBMS-estimated 5-carbon sugars (*p* = 1.55E^−06^) in the native environments and SNP scaffold_14_7970015 was associated with xylose release (*p* = 9.03E^−06^) in Corvallis (Additional file 4, Table 1). Two additional SNPs encompassing a 4.5 kb region surrounding Potri.014G101900 had significant associations with pyMBMS peak *m/z* = 102 (*p* = 8.31E^−05^) in Clatskanie and pyMBMS-estimated 5-carbon sugars (*p* = 5.61E^−05^) in the native environments (Additional files 4 and 5).

A protein kinase Potri.014G142700 located 517 bases upstream of the QTL peak (SNP scaffold_14_10864383) for lignin content identified in the pseudo-backcross pedigree using wood samples from 2-year-old plants (Additional file 3). A SNP marker, scaffold_14_10865898, mapping within Potri.014G142700 ranked first in Clatskanie (*p* = 3.00E^−05^) for association with pyMBMS peak *m/z* = 235, which is derived from methoxyglycolic acid [17]. The same SNP was associated with wet-chemistry estimates for syringyl and guaiacyl monomers (*p* = 7.99E^−05^ and *p* = 9.49E^−05^) in the Surrey environment (Additional file 4, Table 1). A second SNP mapping within the same gene ranked third in the native environments for association with glucose release.

### Protoplast assays

Using a protoplast transient expression assay to activate reporter genes, we tested alternate alleles for three of the six candidate genes—KANADI transcription factor, Potri.014G037200; ANGUSTIFOLIA CtBP, Potri.014G089400; and the protein kinase, Potri.014G142700. The remaining three candidate genes—the amino acid transporter, Potri.014G036500; the copper transport protein ATOX1-related gene, Potri.014G089700; and the Ca^2+^ transporting ATPase, Potri.014G101900, are not suitable for short-lived transient expression assays because they are not known to function at the regulatory level required to evaluate pathway reporter gene activation.

**Figure 2:**
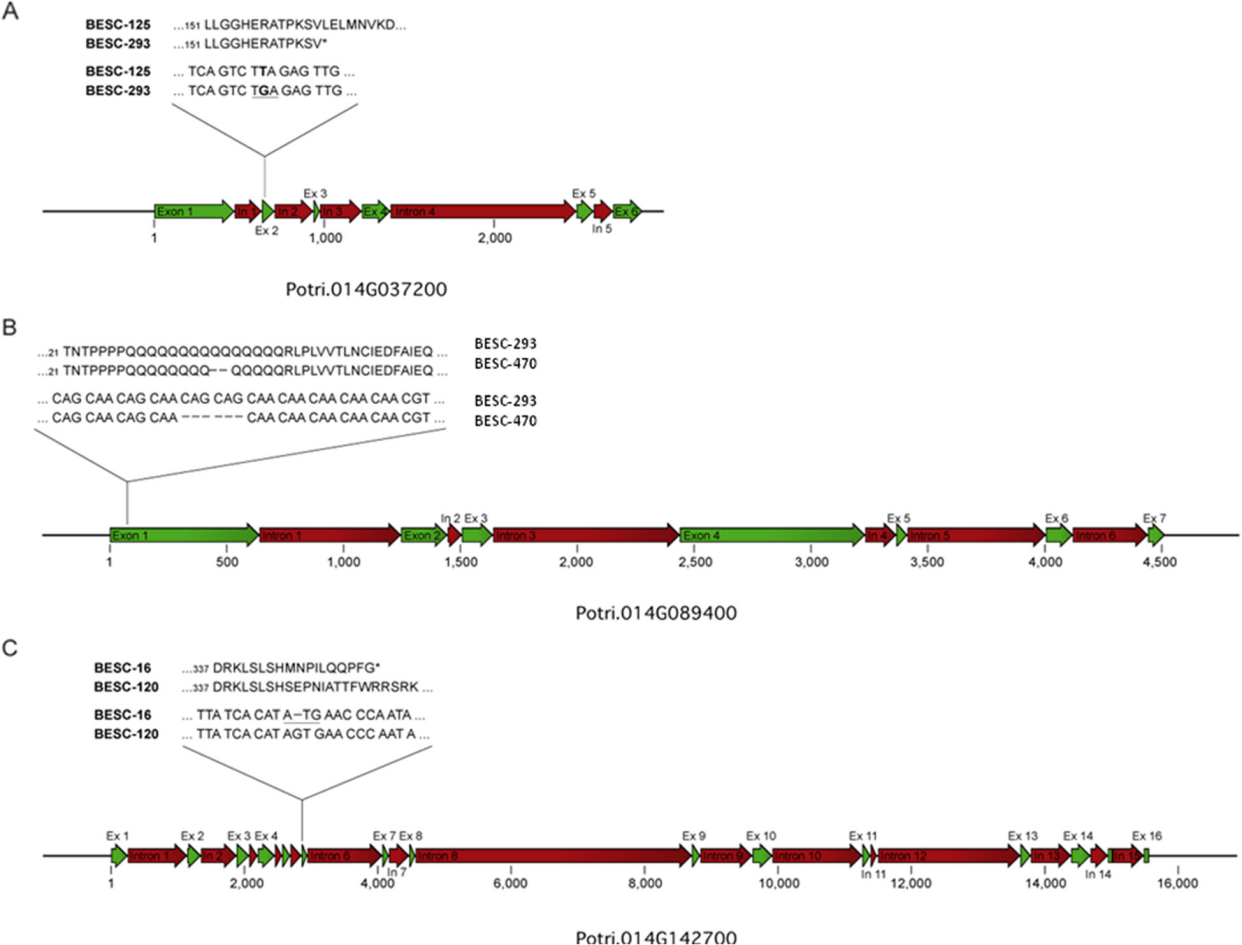
**Partial protein and cDNA alignments of alternate alleles showing positions and effects of polymorphisms in the (A) KANADI transcription factor, Potri.014G037200; (B) Angustifolia CtBP transcription factor, Potri.014G089400; and (C) protein kinase, Potri.014G142700.**

**Figure 3:**
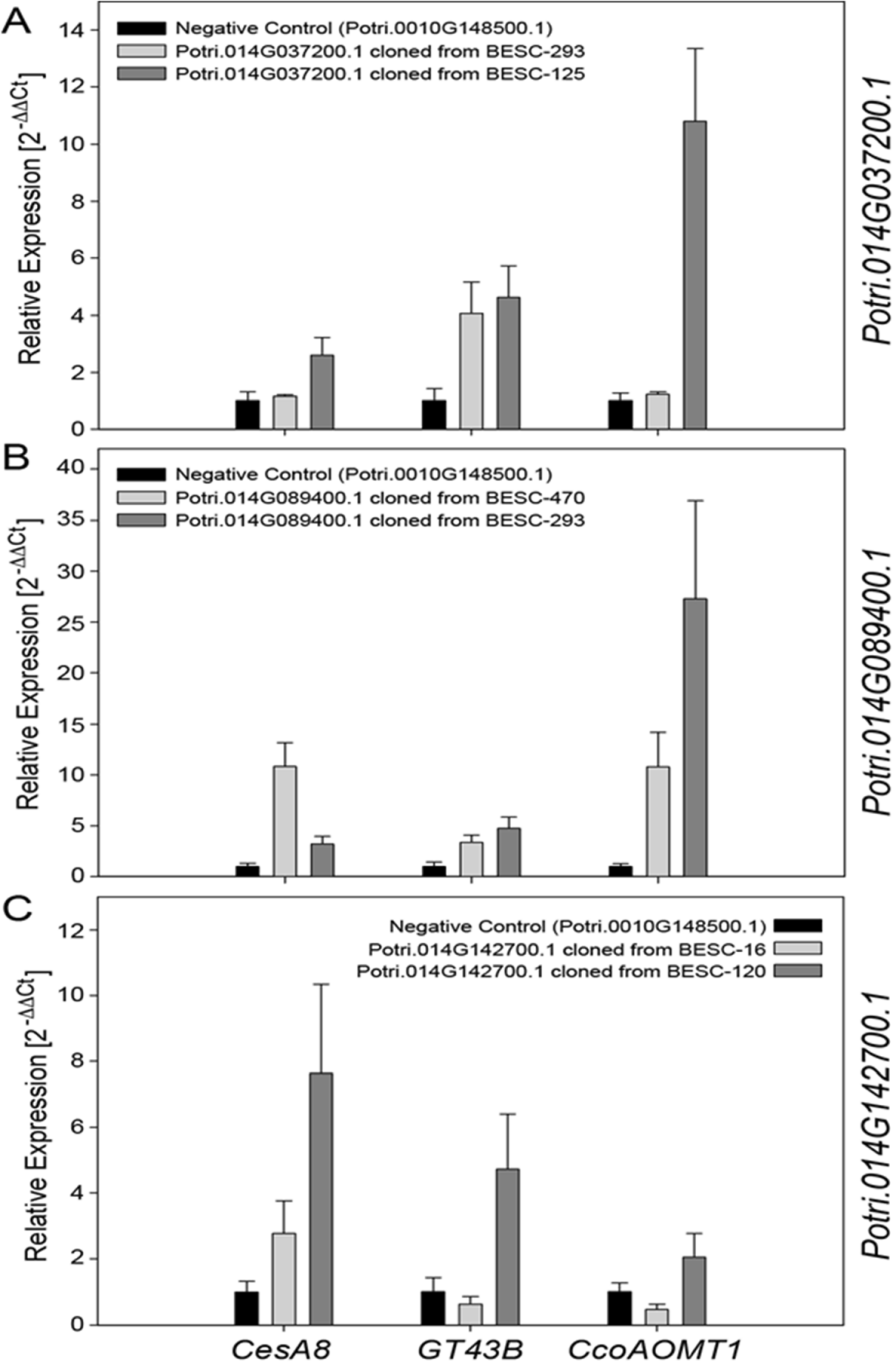
**Differences in activation of reporter genes CesA8 (cellulose), GT43B (hemicellulose) and CCoAOMT1 (lignin) by allelic variants of the (A) KANADI transcription factor, (B) Angustifolia CtBP transcription factor, and (C) protein kinase.** Error bars indicate standard deviations based on three replicates.

Among the alternate alleles for the KANADI transcription factor, Potri.014G037200, there was a single T → G nucleotide substitution at position 496 of the cDNA (Figure 2A) which changed the codon TTA to a TGA premature stop codon leading to a truncated protein of 164 amino acids (Figure 2A). Based on the reference genome protein structure prediction, the MYB-like DNA-binding domain for this gene spans amino acid positions 135 to 186. As such, the allele carrying the premature stop codon was missing 22 amino acids at the C terminus end of the DNA binding domain. The apparent effect of the premature stop codon was supported by the transient expression assay where the truncated protein had markedly lower activation of the lignin biosynthetic pathway reporter gene (CCoAOMT1) compared to the full-length allele (Figure 3A).

Among the alternate alleles for ANGUSTIFOLIA CtBP, Potri.014G089400, there was a tri-nucleotide repeat polymorphism which resulted in the addition of CAGCAG at position 96 from the start codon in one of the alleles. These polymorphisms resulted in two additional glutamine residues in the mature protein. As such, the allele derived from genotype BESC-293 had a longer PolyQ sequence compared to the allele derived from BESC-470 (Figure 2B). In the same allelic variants, there was also a SNP (A/G) polymorphism which resulted in a Thr/Ala amino acid substitution at positions 650 of the longer variant and 648 of the shorter variant (Additional file 6). Results of the transient expression assay suggested that the longer PolyQ/Thr allele had significantly more activation of the CCoAOMT1 reporter gene compared to the shorter PolyQ/Ala allele. The opposite was true when we evaluated activation of the CesA8 reporter gene where the shorter PolyQ allele showed significantly higher activation of the lignin pathway reporter gene (Figure 3B), suggesting that this gene might be involved in concurrent activation/repression of the cellulose and lignin biosynthetic pathway.

Among the alternate alleles for the protein kinase, Potri.014G142700, there was a SNP polymorphism resulting from a single nucleotide indel at position 1,469 of the cDNA which resulted in a premature stop codon at amino acid 355, resulting in a truncated protein (Figure 2C). The predicted functional domain for Potri.014G142700, the protein tyrosine kinase domain, occurring at positions 568 to 813, indicates that the entire kinase domain was absent in the truncated protein. The truncated protein had significantly lower activation of all three reporter genes compared to the allele encoding a full protein (Figure 3C).

### Putative loss-of-function mutations in pseudo-backcross parental genotypes

A key objective of this study was to evaluate co-location of 1) QTLs detected in the pseudo-backcross population and 2) significant association-based SNPs derived from either the Illumina array or resequence-based genotyping data. Within the pseudo-backcross population, we re sequenced the *P. trichocarpa* grandparent ‵93-968′ and the two *P. deltoides* ‘ILL-101′ and ‘D124′ parents and looked for evidence of high-impact mutations such as frameshifts and premature stop codons within the six candidate genes segregating in the BC1 population. Based on this analysis, the ‵93-968′ *P. trichocarpa* genotype carried mutations leading to stop-gained and frame-shifts in three of the six candidate genes. For the KANADI transcription factor, an A → C nucleotide substitution on position 3,030,533 resulted in a premature stop codon. A single nucleotide insertion on position 7,972,460 resulted in a frame-shift mutation within the Ca^2+^ transporting ATPase. In the protein kinase, a single nucleotide insertion on position 10,865,690 resulted in a frame-shift mutation. Mutations in the KANADI transcription factor and protein kinase occurred in the heterozygous state, whereas the mutation in the Ca^2+^ transporting ATPase was homozygous for the allele leading to the frame-shift in the ‵93-968′ genotype. In all three instances, the mutations fell within the intervals delimited by SNPs with the strongest associations across multiple environments described above.

## Discussion

In this study, three different phenotyping platforms were employed to assess variation in cell wall chemistry within *Populus* mapping populations. Of the three, the saccharification assay is markedly different from pyMBMS and traditional cell wall chemistry. This assay measures cell wall recalcitrance by quantifying the amount of monomeric sugars released during cell wall digestion [1-5]. Since these measures tend to vary depending on pretreatment severity, estimates of cell wall composition cannot be confidently inferred from this analysis alone. Alternatively, pyMBMS provides a rapid and high-throughput platform for estimating cell wall composition [18-20]. However, this analysis provides estimates for a limited number of gross-scale measurements, including total lignin, S/G ratio, and 5-carbon and 6-carbon sugars; concerns are often raised about the accuracy of estimates that are based on intensities of a small subset of the hundreds of peaks generated during the analysis [20]. As such, the molecular resolution provided by this assay is limited. On the other hand, traditional cell wall chemistry provides unambiguous quantification of cell wall components and provided quantitative data on up to seventeen different cell wall components in a study by Porth et al. [15]. This method has the disadvantage of being laborious and expensive when it comes to screening large numbers of samples.

Ranges in pyMBMS phenotypes were generally reproducible among alternate sampling years for the pseudo-backcross pedigree and among environments for the association population. Correlations between phenotypes and between sampling years ranged from moderate to very strong. On the other hand, correlations between the various phenotypes and between phenotypes from different environments ranged from not significant to moderately strong. S/G ratio registered the highest correlation between environments with r = 0.31 between plants of varying ages sampled from the native environments and 4-year-old plants in the Clatskanie common garden. In general, glucose release was negatively correlated with lignin content within and between different environments, as well as between phenotypes generated using alternate assays. Significant correlations between different phenotypes corroborate findings from previous studies that can be attributed to two distinct phenomena. First, cell wall recalcitrance is a physical phenomenon resulting from the physical protection of cellulose and hemicellulose fibers by lignin polymers which results in negative correlation between percent lignin content and sugar release during the saccharification process [1-5]. Second, pleiotropy is a molecular genetics phenomenon in which the same genetic loci mediate expression of two different traits. In this regard phenotypes such as lignin content and S/G ratio exhibit high correlations and co-location of QTLs, suggesting the existence of a common set of genes that mediate expression of both traits [6,7].

Our results indicate a genetic basis of cell wall recalcitrance phenotypes in *Populus* and support previous work, including studies employing QTL analysis in interspecific pedigrees [6,7] and LD-based association mapping in diverse *P. trichocarpa* [9,12] and in *P. nigra* [10]. To date, detection of genetic loci based on association mapping has been done mostly in single environments. Our results are the first to show correspondence between loci identified using the two complementary genetic mapping methods across multiple 1) phenotyping and genotyping platforms, 2) environments and growth stages, and 3) years for cell wall traits. This ability to evaluate complex traits across multiple variables provides the much-needed validation of bona fide marker-trait associations in light of often-cited risk of false positives in association mapping studies [8].

There was remarkable overlap between QTLs detected in the pseudobackcross population using samples from age 2 and age 3 wood samples. Specifically, five putative QTLs for S/G ratio and lignin content and three for 5- and 6-carbon sugars were reproducibly identified in this study on LG XIV in the pseudobackcross population. All QTLs exceeded the genome-wise LOD significance in each instance. QTLs for S/G ratio exhibited the highest significance, exceeding LOD scores of 8 in the two independent datasets. In previous studies, Yin et al. reported significant over-representation of cell wall chemistry QTLs on three *Populus* chromosomes, including chromosome XIV, in an F_2_*P. trichocarpa* x *P. deltoides* pedigree, Family 331 [7]. Subsequently, Ranjan et al. reported the genome-anchoring of a subset of these QTLs onto the *Populus* reference genome [21]. Interestingly, a QTL identified and anchored on chromosome XIV in that study co-located with a QTL we identified in the current study using a different mapping population. Ranjan et al. anchored a QTL for xylem S/G ratio encompassing the interval 2,282,351 to 3,052,827 of the v2.2 assembly [21], which corresponds with a QTL peaking at position 2,979,511 (v3.0) for S/G ratio identified in this study. Based on the co-location of QTLs in independent datasets, as well as separate pedigrees, we explored co-location of loci identified using association mapping in two populations of diverse *P. trichocarpa* genotypes.

To this end, we identified 37 significant SNPs mapping within or adjacent to the six candidate genes on chromosome XIV exhibiting reproducible association with cell wall chemistry phenotypes. Remarkably, we detected significant associations for the same candidate genes for related wood chemistry traits measured in drastically different environments and for samples collected from the original source genotype growing in natural stands across a broad latitudinal range. All six genes mapped within the QTL intervals described above. In general, SNPs with significant associations mapped in tight intervals for the six candidate genes across genotyping and platforms, as well as across environments and plant ages. For example, the protein kinase contains a SNP which ranked first in two environments for associations with cell wall chemistry phenotypes characterized on 4- and 9-year-old plants using pyMBMS and wet chemistry, respectively.

Among the six candidate gene loci, an amino acid transporter gene, Potri.014G036500, exhibited the strongest evidence of co-location in both the QTL and association analyses. Two SNPs within 0.71 kb of each other were independently associated with a QTL peak and an association mapping peak. Interestingly, the same QTL interval was also identified in a different mapping population, Family 331, by Ranjan et al. [21]. Amino acid transporters are recognized as key players in lignin biosynthesis since amino acids represent an important form of transportable organic nitrogen [22] and the amino acid phenylalanine is a precursor for lignin biosynthesis [23].

SNPs mapping within or close to a copper transport protein ATOX1-related gene, Potri.014G089700, exhibited reproducible associations across native and common garden environments as well as between phenotyping platforms, ranking in the top 2 associations in the Clatskanie and Surrey environments. SNPs mapping within a 1.6 kb interval ranked first or second for associations with percent lignin, average wood density, and pyMBMS *m/z* = 120 in the two environments and across the two phenotyping platforms. Potri.014G089700 also mapped close to the QTL peak for S/G ratio, which peaked at position 7,068,969. In previous publications, copper has been implicated in lignin biosynthesis in other plant species. Foremost, copper is a well-recognized enzyme cofactor for laccase enzymes whose function has been linked to lignin content in *P trichocarpa* [24]. Other examples implicating copper in lignin biosynthesis include increased shikimate dehydrogenase and peroxidase activity observed in response to elevated copper concentrations leading to enhanced accumulations of phenolics and lignin in *Capsicum annuum* seedling hypocotyls [25]. Kováčik and Klejdus also showed enhanced accumulation of phenolic acids and lignin in copper-treated *Matricaria chamomilla* [26]. In *Pinus radiata*, copper deficiency was associated with poorly lignified wood, leading to deformed trees [27]. Based on these observations, we hypothesize that subcellular copper concentration may play an important role in regulating S/G ratio as well as lignin content. In that regard, cells with reduced copper concentrations may exhibit higher rates of lignin biosynthesis. The predicted function of Potri.014G089700 in copper transportation is supported by multiple repeats of the endoplasmic reticulum-homing dilysine motif (KKEE) [28] found in the predicted protein.

The ANGUSTIFOLIA CtBP gene, Potri.014G089400, which mapped within the same QTL interval and adjacent to the copper transport protein described above, was significantly associated with glucose and xylose release from wood samples collected from both the native environment and the Corvallis common garden. Based on transcript and proteome profiling of developing xylem in *Populus*, Kalluri et al. reported that Potri.014G089400 had enhanced EST expression and protein abundance in xylem tissue [29]. Subsequent cDNA cloning and sequencing using genotypes in our study carrying alternate alleles of the two SNPs revealed a tri-nucleotide CAGCAG repeat polymorphism leading to a PolyQ length polymorphism, as well as a single amino acid substitution between the two alternate alleles. Protoplast assays using the alternate alleles indicated that the allele with the longer PolyQ sequence displayed significantly higher activation of the cellulose pathway reporter gene *CesA8*, but it had lower activation of the lignin pathway reporter gene *CCoAOMT1* compared to the shorter allele. Although the accompanying amino acid substitution cannot be ruled out as causal, effects of variable-length PolyQ stretches on transcription factor activity have been well documented in diverse organisms [30]. In addition, activator/repressor activity of ANGUSTIFOLIA CtBP transcription factor has also been reported in *Arabidopsis*, where the *Arabidopsis* ortholog was shown to regulate leaf-cell expansion, arrangement of cortical microtubules, and the expression of genes involved in cell wall formation [31,32].

cDNA sequencing of the KANADI transcription factor, Potri.014G037200, and the protein kinase, Potri.014G142700, encoding genes, each having support from multiple environments and alternate genetic mapping experiments, revealed a single nucleotide substitution and a single nucleotide deletion, respectively, that resulted in premature stop codons in both transcripts. In each case, the functional domain for each gene was partially or fully lost in the truncated proteins. Transient expression assays for the protein kinase suggested reduced activation of both the cellulose and the hemicelluloses pathways by the allele harboring the premature stop codon. Similarly, the allele harboring the premature stop codon in the KANADI transcription factor had markedly reduced activation of the lignin biosynthetic pathway. Based on gene family classification of the *Arabidopsis* ortholog At5g42630, this transcription factor belongs to the KANADI family of transcription factors, which has been implicated in spatial arrangement of phloem, cambium, and xylem [33].

Although the specific influence of the Ca^2+^ transporting ATPase gene, Potri.014G101900, on cell wall biosynthesis will require further validation, there were robust associations between SNPs within Potri.014G101900 and 5- and 6-carbon sugars across different environments. The importance of calcium in modulating cell wall properties is well recognized [34,35]. Specifically, Lautner et al. demonstrated the favorable effect of calcium nutrition on wood formation in *Populus* using *P. tremula* x *P. tremuloides* [35]. In this study, Lautner et al. reported a significant reduction of the syringyl units of lignin under calcium starvation as well as an increase in fiber length with increasing Ca^2+^ nutrition [35].

## Conclusions

Given that map-based cloning of candidate genes is extremely time and resource intensive, even for model species with abundant resources, the possibility of using association mapping as a tool for cloning genes of functional relevance has the potential to accelerate genetic improvement of the complex perennial *Populus*. In this study, we have demonstrated the power of complementary genetic mapping approaches (i.e., QTL- and association-based) to identify genes affecting cell wall recalcitrance across diverse genetic backgrounds, sampling environments, and phenotyping and genotyping platforms. Using this combinatorial approach, we identified genes that have not been previously linked to cell wall recalcitrance in *Populus*. Although the physiological impact of calcium, copper, and amino acid availability on cell wall structure has been previously described, the genes involved in transportation (and thus the putative availability of these metal ions and lignin precursors to developing cell walls) provide hitherto unexplored targets for modulating cell wall biosynthesis. Using the *Populus* protoplast transient expression assay, we obtained supportive evidence for three genes identified by our genetic approach as potential novel regulators of the expression of major cell wall biosynthesis enzymes. Using this *in vivo* system to confirm function of the putative causal alleles was instructive in dissecting the potential biological roles of beneficial alleles.

We have also demonstrated the potential value of natural variants as a source and platform for molecular studies in plants. Five of the six genes identified in this study had sequence-based evidence of naturally occurring loss-of-function mutations. Natural variants could serve as an important resource for studying of gene function in systems such as *Populus* in which genetic manipulation can be cumbersome. In breeding programs, genotypes carrying loss-of-function and enhancer mutations could be strategically used in marker-assisted breeding schemes to pyramid complementary mutations that might result in superior phenotypes, similar to the ‘breeding with rare defective alleles’ (BRDA) approach illustrated by Vanholme et al. [36]. Finally, the apparent enhancement of transcription factor activity by the PolyQ stretch suggests the possibility of using naturally heightened activity as well as opportunities to engineer multiple tandem PolyQ segments for enhanced versions of transcription factors regulating the expression of genes affecting economically important traits.

## Methods

### Plant materials

#### QTL mapping pedigree

A pseudo-backcross population with 712 individuals was established in a replicated field trial in Morgantown, WV (39°39′32″N 79°54′19″W). The trial consisted of two clonal replicates in an interlocking block design [37]. Genotypes were initially planted at 2 m × 2 m spacing using rooted cuttings in June 2007. The trial was thinned by removing 50% of clones in a diamond fashion in December 2008 (age 2), leaving plants at 2.83 m × 4 m spacing. The remaining genotypes were harvested in January 2010 (age 4).

#### Association mapping populations

A population of 1,089 black cottonwood genotypes (*P. trichocarpa*) was collected from native stands to encompass the central portion of the natural range of the species, stretching from 38.8° to 54.3° N latitude from California to British Columbia [13], hereafter referred to as the BESC population. Wood samples for this study were collected from the native ortets in addition to clonally replicated wood samples from field sites in Corvallis, OR (44°34′14.81″N 123°16′33.59″W) (age 2) and Clatskanie, OR (46°6′11″N 123°12′13″W) (age 4). A partially overlapping and independently phenotyped population of 499 *P. trichocarpa* genotypes was assembled from 44° to 58.6° N and established in Surrey, British Colombia, as described by Porth et al. [15]. The population shared 146 genotypes with the BESC population. Wood samples for phenotyping were collected from the Surrey plantation from 9-year-old trees. Details about the establishment, sampling and phenotyping of the mapping pedigree and association populations are given in Additional file 2.

Although overlap in genotypes was observed across multiple locations, differences in age at sampling represented an additional variable with significant contribution in phenotypic expression. As such, each defined environment was taken to represent the physical and climatic conditions as well as the developmental stage of the plants at each site.

### Phenotyping

Wood disks cut from each stem 1.2 m off the ground for each genotype in the pseudo-backcross mapping pedigree were collected in December 2008 and February 2010 from 2- and 3-year-old plants, respectively. In January 2008, 4.3 mm increment cores were collected from 570 of the 1,100 ortet *P. trichocarpa* genotypes in their native environments in the core of the range (from the Columbia River in northern Oregon to the Skagit River in northern Washington). In December 2010, 300 single-replicate stem disks were harvested from 2-year-old plants in Corvallis, OR. In June 2012, 4.3 mm increment cores were collected from 932 4-year-old plants in Clatskanie, OR. Of these 932 genotypes, 235 had two clonal replicates. Debarked and air-dried increment cores and stem disks were ground using a Wiley Mini-Mill (Swedesboro, NJ) with a 20-mesh screen. Lignin content, S/G ratio, and 5- and 6-carbon sugar content were determined by pyMBMS analysis, and glucose and xylose release were characterized based on the saccharification assay. These assays were conducted at the National Renewable Energy Laboratory (Golden, CO) as described below. The Surrey population was characterized for 17 cell wall traits using traditional wet chemistry assays at the University of British Colombia, Vancouver, BC, Canada as described in Porth et al. [15].

For the BESC population, 300, 797, and 926 genotypes were available for analyses in Corvallis, native, and Clatskanie environments, respectively. After eliminating genotypes with evidence of sibship [12] and missing SNP data >10%, the BESC and Surrey populations shared 146 genotypes for phenotypic correlation analysis and 123 genotypes for association mapping analysis.

### Saccharification

Wood samples were treated with α-amylase (spirizyme Ultra—0.25%, Novozymes, North America, Inc., Franklinton, NC) and β-glucosidase (Liquozyme SC DS—1.5%, Novozymes) in 0.1 M sodium acetate (24 h, 55°C, pH 5.0) to remove starch (16 ml enzyme solution per 1 g biomass). This was followed by Soxhlet extraction in ethanol (95% v/v) for 24 h to remove extractives. After overnight drying, 5 mg (±0.5 mg) of extractives-free biomass was weighed in triplicate into a solid Hastelloy 96-well microtiter plate. 250 μl H_2_O were added, and samples were sealed with silicone adhesive and Teflon tape and heated at 180°C for 40 min. Once cooled, 40 μl of buffer-enzyme stock was added. The buffer-enzyme stock consisted of 8% CTec2 (Novozymes) in 1 M sodium citrate buffer. The samples were then gently mixed and left to statically incubate at 50°C for 70 h. After the 70 h incubation, an aliquot of the saccharified hydrolysate was diluted and evaluated using the glucose oxidase/peroxidase and xylose dehydrogenase assays (Megazyme International Ireland, Wicklow, Ireland). Results were calculated using calibration curves constructed from standard mixtures of glucose and xylose.

### Pyrolysis MBMS

A commercially available MBMS designed specifically for plant biomass analysis was used for pyrolysis vapor analysis [18-20]. Briefly, approximately 4 mg of air-dried 20 mesh biomass was introduced into a quartz pyrolysis reactor via 80 μl stainless steel Eco-Cups provided with the autosampler. Mass spectral data from 30–450 *m/z* were acquired on a Merlin Automation data system version 3.0 using 17 eV electron impact ionization.

Lignin estimates were determined by summing the intensities of peaks assigned to lignin compounds as described by Sykes et al. [20]. The lignin intensities were then corrected to a standard with a known Klason lignin content using a single point correction technique. S/G ratios were determined by summing the peaks ascribed to syringyl moieties (namely, *m/z* 154, 167, 168, 182, 194, 208, and 210) and dividing by the sum of peaks ascribed to guaiacyl-derived units (*m/z* 124, 137, 138, 150, 164, and 178).

Sugar estimates were determined by summing the intensities of peaks assigned to 5- and 6- carbon sugars from model compound experiments [18]. 5-carbon sugars were determined by summing peak intensities of *m/z* 57, 73, 85, 96, and 114, while 6-carbon sugars were estimated by summing peak intensities *m/z* 57, 60, 73, 98, 126, and 144.

### SNP genotyping of the ‵52-124′ pedigree and map construction

712 pseudo-backcross progeny and parental lines were genotyped using a 5 K Illumina Infinium SNP array (Illumina, San Diego, CA) containing 5,390 probes. Details of array design, target SNP selection, and SNP analysis and genetic map construction are given in Additional file 2.

### Illumina Infinium SNP genotyping

The 34 K Illumina Infinium® SNP array described by Geraldes et al. [14] was used to genotype 991 and 334 individuals of the BESC and Surrey populations, respectively.

### Whole-genome resequencing

A second genotyping platform based on whole-genome resequencing was used to characterize SNP and indel polymorphisms in 673 of the 1,089 genotypes. Details of this analysis are provided in Additional file 2.

### QTL mapping

A maximum likelihood inference method implemented in the Multiple-QTL Mapping (MQM) package of MapQTL 6.0 [38] was used to identify QTLs within the pseudo-backcross population. One thousand permutations were conducted separately for each trait and experiment to determine genome-wise LOD significance threshold at *p* ≤ 0.05 [39]. QTLs were declared significant when identified (i.e., having LOD scores above the significance threshold) in at least two independent experiments or between two different phenotypes in the same experiment. A drop in LOD score of 1.0 was used to declare adjacent QTLs as separate loci.

### Association mapping based on Infinium array data

Based on evidence of a major QTL hotspot for cell wall phenotypes, SNPs distributed across chromosome XIV of the assembly were specifically evaluated for association with recalcitrance phenotypes. SNPs with a MAF ≥0.05 from the Infinium array and resequencing data were used in this part of the study. Firstly, SNP trait associations were evaluated for the Infinium array on a whole chromosome scale by including all SNPs regardless of QTL information. Secondly, we extracted SNPs lying within the ±1 LOD intervals delimiting individual QTLs and performed a QTL scale analysis. TASSEL 3.0 (http://www.maizegenetics.net) was used to identify marker-trait associations based on the mixed linear model analysis using kinship (K) to define genetic covariance among genotypes and population structure (Q) as a covariate [40]. Cell wall chemistry phenotypes, as well as individual *m/z* peak intensities from the pyMBMS analysis, were analyzed. Descriptions of cell wall phenotypes can be found in Additional file 2.

### Association mapping based on resequencing data

Candidate gene intervals identified based on the Infinium array data were saturated with SNPs from the resequencing effort and reanalyzed for associations using phenotypic data from Corvallis, Clatskanie, and native environments. Candidate intervals were saturated by selecting SNPs within each candidate gene plus 10 kb flanking regions.

### Statistical analysis

Correction for multiple testing was conducted using the unadjusted Bonferroni correction [41] on a chromosome-wise level using all SNP markers and on a QTL-interval-wise level employing SNPs falling within QTL and candidate gene intervals. Spearman’s rank correlation analyses were performed using the Statistix 8 software [42].

### cDNA cloning and *Populus* protoplast transient expression assay

Regulatory genes including two transcription factors and a protein kinase, whose activity could be measured relative to activation of reporter genes, were selected for cloning and use in a transient expression assays. Genotypes carrying alternate alleles were used to clone two allelic variants of each gene for side-by-side comparison relative to an empty vector control using the protoplast transient expression assay as described in Additional file 2. cDNA sequences from alternate alleles were deposited under accession numbers Potri.014G037200_BESC-125 [GenBank:KP271989], Potri.014G037200_BESC-293 [GenBank:KP271990], Potri.014G089400_BESC-470 [GenBank:KP271991], Potri.014G089400_BESC-293 [GenBank:KP271992], Potri.014G142700_BESC-16 [GenBank:KP271993], Potri.014G142700_BESC-120 [GenBank:KP271994]. Additional files 7, 8, 9 and 10 provide supporting details for materials and methods and results described in Additional file 2.

### Availability of supporting data

SNP data is available from the Joint Genome Institute’s Phytozome database (http://genome.jgi-psf.org/pages/dynamicOrganismDownload.jsf?organism=Ptrichocarpa).

## Additional files

Additional file 1:
**Phenotypic correlations.**
Additional file 2:
**Material and methods.**
Additional file 3:
**QTL mapping results.**
Additional file 4:
**34 K Infinium array-based association mapping results.**
Additional file 5:
**Whole-genome resequencing-based association mapping results.**
Additional file 6:
**Protein alignment showing allelic variants of the Angustifolia CtBP transcription factor Potri.014G089400.**
Additional file 7:
**Validation of the constructed transient over-expression vector using the** ***GUS*** **reporter assay.**
Additional file 8:
**Validation of the feasibility of using** ***Populus*** **protoplasts as a tool to study regulation of reporter genes for the cellulose, hemicellulose and lignin biosynthesis pathways.**
Additional file 9:
**Primer list.**
Additional file 10:
**Phenotypic variation in mapping populations.**
