# Supplementary material for: High-resolution genetic mapping of allelic variants associated with cell wall chemistry in Populus
Source: BMC Genomics. 2015 Jan 23;16(1):24. doi: 10.1186/s12864-015-1215-z (PMC4307895; doi:10.1186/s12864-015-1215-z)
Supplement: Additional file 1: — Phenotypic correlations. [file 12864_2015_1215_MOESM1_ESM.docx]

Additional File 1A. Spearman’s rank correlations for cell-wall chemistry traits determined by pyMBMS on *P. trichorcapa* x *P. deltoides* pseudo-backcross progeny (*n*=515). Significant correlations between years are shown in bold (*p*<0.05), ns = not significant.

| Traits | 5-carbon sugars_2^nd^ year | 6-carbon sugars_2^nd^ year | % lignin_2^nd^ year | % lignin_3^rd^ year | S/G ratio_2^nd^ year |
| --- | --- | --- | --- | --- | --- |
| 6-carbon sugars_2^nd^ year | 0.9103 |  |  |  |  |
| % lignin_2^nd^ year | -0.6514 | -0.7697 |  |  |  |
| % lignin_3^rd^ year | **-0.6162** | **-0.7271** | **0.9288** |  |  |
| S/G ratio_2^nd^ year | 0.0096ns | -0.1120 | 0.3698 | **0.3333** |  |
| S/G ratio_3^rd^ year | 0.0167ns | **-0.0995** | **0.3302** | 0.3569 | **0.9367** |

Additional File 1B. Spearman’s rank correlations for pyMBMS and saccharification phenotypes among diverse *P. trichocarpa* genotypes sampled from their native environments,

Clatskanie, and Corvallis common gardens (*n*=258). Statistically significant correlations across different environments are shown in bold (*p*<0.05), ns = not significant.

| Traits | % lignin_Clatskanie | S/G ratio_Clatskanie | % lignin_Corvallis | S/G ratio_Corvallis | % lignin_Native |
| --- | --- | --- | --- | --- | --- |
| S/G ratio_Clatskanie | 0.4905 |  |  |  |  |
| % lignin_Corvallis | **0.2023** | **0.2397** |  |  |  |
| S/G ratio_Corvallis | 0.1174ns | **0.4258** | 0.3559 |  |  |
| % lignin_Native | **0.1424** | **0.1808** | -0.0402ns | 0.1132ns |  |
| S/G ratio_Native | -0.0131ns | **0.3378** | 0.1173ns | **0.3083** | 0.0895ns |

Additional File 1C. Spearman’s rank correlations for pyMBMS and saccharification phenotypes in diverse *P. trichocarpa* genotypes sampled from their native environments and the Clatskanie common garden (*n*=795). Statistically significant correlations across different environments are shown in bold (*p*<0.05), ns = not significant.

| Traits | Glucose release_Clatskanie | Glucose/xylose release_Clatskanie | Xylose release_Clatskanie | % lignin_Clatskanie | S/G ratio_Clatskanie | Glucose release_Native | Glucose/xylose release_Native | Xylose release_Native | % lignin_Native | S/G ratio_Native | 5-carbon sugars_Native |
| --- | --- | --- | --- | --- | --- | --- | --- | --- | --- | --- | --- |
| Glucose/xylose release_Clatskanie | 0.8738 |  |  |  |  |  |  |  |  |  |  |
| Xylose release_Clatskanie | 0.0299ns | 0.4724 |  |  |  |  |  |  |  |  |  |
| % lignin_Clatskanie | -0.3943 | -0.1740 | 0.3564 |  |  |  |  |  |  |  |  |
| S/G ratio_Clatskanie | -0.1523 | -0.0198ns | 0.2247 | 0.4792 |  |  |  |  |  |  |  |
| Glucose release_Native | -0.0457ns | 0.0347ns | **0.1600** | -0.0445ns | **0.1211** |  |  |  |  |  |  |
| Glucose/xylose release_Native | -0.0143ns | 0.0366ns | **0.1415** | -0.0525ns | **0.1415** | 0.9702 |  |  |  |  |  |
| Xylose release_Native | **-0.0789** | -0.0065ns | **0.1383** | -0.0081ns | 0.0718ns | 0.7431 | 0.5821 |  |  |  |  |
| % lignin_Native | -0.0433ns | -0.0172ns | 0.0653ns | **0.1432** | **0.0980** | -0.1626 | -0.2124 | 0.0285ns |  |  |  |
| S/G ratio_Native | -0.0340ns | 0.0272ns | **0.1286** | 0.0451ns | **0.3088** | 0.5729 | 0.5280 | 0.5394 | 0.0976 |  |  |
| 5-carbon sugars_Native | -0.0143ns | 0.0306ns | **0.0927** | -0.0535ns | 0.0625ns | 0.4403 | 0.4477 | 0.2817 | -0.7014 | 0.3517 |  |
| 6-carbon sugars_Native | 0.0312ns | 0.0230ns | -0.128ns | **-0.1153** | -0.0178ns | 0.2817 | 0.3198 | 0.0745 | -0.8846 | 0.1196 | 0.8736 |

Additional File 1D. Statistically significant correlations (*p*<0.05) for cell wall chemistry phenotypes in diverse *P. trichocarpa* genotypes measured using different assays. pyMBMS and saccharification was performed on samples from trees in their native environments and 4-yr-old trees in the Clatskanie common garden. Wet chemistry assays were performed on samples from 9-yr-old trees from the Surrey common garden (*n*=146).

| pyMBMS/Saccharification phenotype | Wet chemistry phenotype | *r _s_* |
| --- | --- | --- |
| S/G ratio_Native | Xylose | 0.1677 |
| S/G ratio_Native | Hemicellulose/total lignin | 0.1678 |
| S/G ratio_Native | Total Lignin | -0.1855 |
| S/G ratio_Native | S/G-monomers | 0.6083 |
| S/G ratio_Native | S-monomers | 0.6083 |
| S/G ratio_Native | Alphacellulose/total lignin | 0.2194 |
| S/G ratio_Native | Soluble lignin | 0.3910 |
| S/G ratio_Native | Insoluble lignin | -0.2969 |
| S/G ratio_Native | Alphacellulose | 0.1877 |
| S/G ratio_Clatskanie | Total lignin | 0.2517 |
| S/G ratio_Clatskanie | S/G monomers | 0.5521 |
| S/G ratio_Clatskanie | S-monomers | 0.5521 |
| S/G ratio_Clatskanie | Soluble lignin | 0.2045 |
| S/G ratio_Clatskanie | Arabinose | -0.2505 |
| S/G ratio_Clatskanie | Alphacellulose/hemicelluloses | 0.1802 |
| Glucose release_Native | Xylose | 0.1945 |
| Glucose release_Native | Hemicellulose/total lignin | 0.1771 |
| Glucose release_Native | Total lignin | -0.2369 |
| Glucose release_Native | S/G monomers | 0.1735 |
| Glucose release_Native | S monomers | 0.1735 |
| Glucose release_Native | Alphacellulose/total lignin | 0.2302 |
| Glucose release_Native | Insoluble lignin | -0.2488 |
| Glucose/xylose release_Native | Xylose | 0.1636 |
| Glucose/xylose release_Native | Total lignin | -0.2370 |
| Glucose/xylose release_Native | Alphacellulose/total lignin | 0.2057 |
| Glucose/xylose release_Native | Insoluble lignin | -0.2354 |
| Xylose release_Native | Xylose | 0.2049 |
| 5-carbon sugars_Native | Xylose | 0.1721 |
| 6-carbon sugars_Native | Hemicellulose/total lignin | 0.1980 |
| Percent lignin_Native | Xylose | -0.1647 |
| Percent lignin_Native | Hemicellulose/total lignin | -0.1862 |
