# Supplementary material for: High-resolution genetic mapping of allelic variants associated with cell wall chemistry in Populus
Source: BMC Genomics. 2015 Jan 23;16(1):24. doi: 10.1186/s12864-015-1215-z (PMC4307895; doi:10.1186/s12864-015-1215-z)
Supplement: Additional file 2: — Material and methods. [file 12864_2015_1215_MOESM2_ESM.docx]

**Materials and Methods**

**QTL mapping pedigree:** The population was developed by crossing a female *P. trichocarpa* clone, ‘93-968’ from western Washington state, with a male *P. deltoides* clone, ‘ILL-101’ from southern Illinois. The female F_1_ genotype, ‘52-225,’ was crossed with an alternate male *P. deltoides* clone from Minnesota, ‘D124,’ to create the 52-124 pseudo-backcross population.

**Association mapping populations:** The BESC population was collected from native stands to encompass the central portion of the natural range of the species, stretching from 38.8° to 54.3° N latitude from California to British Columbia (1), hereafter referred to as the BESC population. Propagation materials were collected from individual ortets, clonally replicated under nursery conditions at Mount Jefferson Farms, Salem, OR, in 14 cm cone-tainers (Stuewe and Sons, Corvallis, OR) and subsequently established in replicated field plots in Placerville, CA (38°43′47″N 120°47′55″W); Corvallis, OR ([44°34′14.81″N 123°16′33.59″W](http://tools.wmflabs.org/geohack/geohack.php?pagename=Corvallis%2C_Oregon&params=44_34_14.81_N_123_16_33.59_W_type:city_region:US-OR)); and Clatskanie, OR (46°6′11″N 123°12′13″W). A partially overlapping and independently phenotyped population of 499 *P. trichocarpa* genotypes was collected from a latitudinal range spanning from 44° to 58.6° N and established in Surrey, British Colombia. as described by Porth et al. (2).

**5K Illumina Infinium SNP array design and genetic map construction:** We used whole genome resequencing data (1) for the parent trees of pedigrees to select SNP loci for an Illumina Infinium BeadArray assay. We selected loci that were homozygous for one allele in clone 93‑968 and homozygous for a different allele in clones ILL-101 and D-124. This ensured that the loci would be heterozygous in clone 52-225 (which was not sequenced) and that at least one allele would segregate in family 52-124. We further required that there were no additional polymorphisms in any of the resequenced trees within 30 bp of the target polymorphism and that the depth of sequence was at least 12 across the region. From among the loci for which an Infinium II assay could be designed, we selected 5,031 loci with even spacing across the 19 chomosomes in the v2.2 *Populus* assembly, as well as 969 loci on unassembled scaffolds. Of these, 5, 390 SNP probes were successfully designed

SNP clusters were visualized using the Illumina GenomeStudio software V2010.3 (Illumina, CA), and they were manually curated for cluster separation before extracting genotype calls. SNPs with the expected segregation pattern, a minimum GenTrain score of 0.15, and nonoverlapping clusters were considered for downstream analysis. Genotypes with more than 10% missing data were excluded from any analysis. Genetic mapping was conducted using JoinMap 4 software (3). After exclusion of progeny with more than 5% missing data and identical clones, 692 of the 712 individuals were used in map construction using 3,751 segregating SNP markers with 0.3 minor allele frequency (MAF). Markers heterozygous in the F_1_ parent and homozygous in the deltoides parents were analyzed using the cross pollination population type. Linkage groups were defined using a minimum independence LOD threshold of 100 and 0.45 recombination frequency. Marker order and cM distances within linkage groups were determined using the using the regression algorithm and Haldane’s mapping function, respectively, with default options. Linkage groups were numbered according to markers derived from the 19 chromosome-scale scaffolds in the *P. trichocarpa* whole-genome assembly (4).

***Populus* 34K Illumina Infinium SNP genotyping.** The 34K array (5) was designed to encompass SNPs distributed in and around 3,543 genes, and it was based on v2.2 of the *Populus* reference genome (http://www.phytozome.net/cgi-bin/gbrowse/poplar/). SNP data were visualized and curated as described above. SNP positions for the 5K and 34K Infinium arrays were translated into v3.0 positions by aligning sequences flanking the SNP against the v3.0 assembly (http://www.phytozome.net/cgi-bin/gbrowse/poplar/). SNP names included the scaffold number followed by the physical position of the SNP.

**Whole-genome re-sequencing.** We obtained plant materials from 673 black cottonwood (*Populus trichocarpa* Torr & Gray) from the central latitudinal range of the species as previously described (1). DNA was extracted from greenhouse-grown plants using a Qiagen kit (Valencia, CA) and quantified using a picogreen assay. Whole genome resequencing to at least 15x depth was done at the Joint Genome Institute using Illumina Genome Analyzer and HiSeq200 instruments (Illumina, CA). Short reads were then aligned to the *P. trichocarpa* version 3 genome using BWA 0.5.9-r16 backtrack algorithm (aln + sampe) with default parameters (6). The resulting bam was further processed to correct mate pair flags and mark duplicate molecules using the FixMateInformation and MarkDuplicates methods in the Picard package (<http://picard.sourceforge.net>). SNPs and small indels for the merged dataset were called using SAMtools mpileup (-E –C 50 –DS –m 2 –F 0.000911 –d 50000) and bcftools (-bcgv –p 0.999089) (7).

**Population structure and kinship.** Q estimates of population structure were computed based on a set of 1507 SNPs with no missing data and MAF ≥0.05 distributed across the 19 scaffolds of the genome assembly. The admixture model with correlated allele frequencies was run in the software STRUCTURE 2.3.3 with 10,000 burnins and 10,000 MCMC replications after burnin for K=1 to 15. The *K* estimate with the highest mean ln P(D) value was accepted as the number of distinct subpopulations. A pairwise kinship matrix was generated based on 27,940 SNPs with ˂10% missing data and MAF ≥0.05 using Tassel 3.0 software.

**Vector construction.** A Gateway compatible construct for transient gene expression in protoplast was made by first digesting pSAT4A-DEST-n(1-174)EYFP-N1 (ABRC stock #CD3-1080) and pSAT5-DEST-c(175-end)EYFP-C1(B) (ABRC stock #CD3-1097) (8) with NdeI and BglII, then ligating the 1.1 kb fragment of the first construct and 4.4 kb fragment of the second one. The efficacy of this construct was validated by over-expressing a *GUS* gene in protoplasts (Additional File 7). The coding sequence of each *Populus* gene was cloned from cDNA by PCR. The DNA fragments were introduced into a pENTR vector by using a pENTR™/D-TOPO® Cloning Kit (Invitrogen). The gene of interest was then subcloned into the above-mentioned expression construct using LR clonase (Invitrogen).

**cDNA cloning and protoplast assays.** First, we demonstrated the efficacy of the assay by using transcription factors PtrWND2B and PtrMYB20, which were previously shown to induce the expression of marker genes, and comparing them to empty-vector controls (Additional File 8). Then, greenhouse-grown genotypes from the 1,100 *P*. *trichocarpa* association population carrying alternate alleles of target genes were used to clone cDNAs for the protoplast assay using primers listed in Additional File 9. Sequence verification was done by sequencing each cDNA from both directions. Sequence translation was done using the ExPASy online translation tool (http://web.expasy.org/translate/), and cDNA and protein alignments were generated using the online EMBL-EBI ClustalW2 tool (http://www.ebi.ac.uk/Tools/msa/clustalw2/).

Alternate alleles at each candidate locus (as well as a negative control, the Cas1p-like gene Potri.010G148500) were transfected into *Populus* protoplasts and evaluated for the induction of marker genes for cellulose, hemicelluloses, and lignin biosynthetic pathways described below. The *Populus* protoplast transfection assay was conducted as described by Guo et al., (9). Briefly, intact protoplasts were isolated from leaves of the *Populus* genotype 717 cultured on MS medium in a Magenta box (9). Protoplasts from the same isolation were separated into three pools for side-by-side transfection with two alternate alleles and the negative control. Each transfection treatment was replicated three times. Transfected protoplasts were incubated overnight under low light condition (10 µmol s^-1^m^2^) to facilitate the expression of the transgene. Total RNA was extracted from approximately 5 million protoplasts with Trizol (Invitrogen Inc., Carlsbad, CA). Two-hundred-fifty microliters of Trizol was used for each RNA extraction, and linear polyacrylamide (10) was added in the RNA precipitation step as a carrier. 500 ng of total RNA was used for reverse transcription using RevertAid^TM^ Reverse Transcriptase (Fermentas Inc. Hanover, MD, USA) and oligo dT_16_ as the primer. The real-time PCR (RT-PCR) primers listed in Additional File 9 were designed using the NCBI Primer-BLAST tool (11). The specificity of each primer pair was determined by aligning the primers against the reference RNA sequence database for *P*. *trichocarpa* (http://blast.ncbi.nlm.nih.gov/Blast.cgi) using blastn. RT-PCR reactions were conducted on a StepOne Plus^TM^ RT-PCR system (Applied Biosystems) with the iTaq^TM^-SYBRH Green Super Mix with ROX (Bio-RAD Inc.). Expression of the *Populus* ubiquitin gene, Potri.001G418500, was used to standardize the expression of each gene. A 35S::GFP (ABRC stock #: CD3-911) construct was co-transfected for each sample to monitor the transfection efficiency in each assay. Only assays with estimated transfection efficiency of 60% or higher were used for qRT-PCR analysis.

The expression of three marker genes associated with cell biosynthesis pathways [*PtrCesA8* (Potri.011G069600) for cellulose, *PtrGT43B* (Potri.016G086400) for hemicellulose, and *PtrCCoAOMT1* (Potri.009G099800) for lignin biosynthesis (12)] was used to assess differences in activation potential among allelic variants and the negative control. Two transcriptional factors known to regulate the expression of the three marker genes, PtrWND2B (13) and PtrMYB20 (14), were used to validate this system (9). In order to construct the promoter::GUS reporter, the 2 kb sequence upstream of the complete coding sequences (CDS) of the three reporter genes was cloned and fused to a *GUS* gene by replacing the *UBQ10* promoter of the HBT95-pUBQ10-GUS construct reported previously (15).

**Results**

pyMBMS analysis of the *P. trichocarpa* x *P. deltoides* pseudo-backcross population

Lignin content within the pseudo-backcross ranged from 21.8 to 30.7% in 2008 and 23.2 to 32.7% in 2010 among the 2- and 3-yr-old plants. S/G ratios for the same materials ranged from 1.5 to 2.5 in both of the datasets. Peak intensities of 5- and 6-carbon sugars were only evaluated in the 2008 sampling, and phenotypic values ranged from 23.7 to 34.4 and 24.8 to 36.7 for total 5 and 6-carbon sugars, respectively (Additional File 10).

**pyMBMS analysis of the *P. trichocarpa* population.** The lowest lignin contents within the native, Corvallis, and Clatskanie environments were 15.7, 20.6, and 17.7%, while the highest lignin values were 27.9, 28.0, and 28.1%, respectively (Additional File 10). S/G ratios ranged from 1.0 to 3.0 in the native environments, between 1.5 and 2.4 in the Corvallis-grown plants, and between 1.3 and 2.5 in the Clatskanie-grown plants. 5 and 6-carbon sugar peak intensities in the native environments ranged from 18.1 to 29.9 and 21.8 to 43.2, respectively. In Corvallis, the same phenotypic values ranged from 19.5 to 31.7 and 20.3 to 38.3, respectively.

**Saccharification analysis of the *P. trichocarpa* population.** Glucose release ranged from 0.01 to 0.48 mg/mg biomass in the native environments, 0.01 to 0.21 in Corvallis, and 0.17 to 0.50 in Clatskanie. Xylose release for the same environments ranged from 0.07 to 0.19 mg mg^-1^ biomass, 0.01 to 0.19, and 0.09 to 0.24 mg mg^-1^ biomass, respectively (Additional File 10). Glucose release was negatively correlated with lignin content in both native and Clatskanie environments as well as between the native environments and the Surrey populations that were phenotyped using different platforms (Additional File 1D).

**References**

1. Slavov GT, DiFazio SP, Martin J, Schackwitz W, Muchero W et al. Genome resequencing reveals multiscale geographic structure and extensive linkage disequilibrium in the forest tree *Populus trichocarpa*. *New Phytologist* 2012, 196:713-725.
2. Porth I, Klápště J, Skyba O, Lai BSK, Geraldes A, Muchero W, Tuskan GA, Douglas CJ, El-Kassaby YA, Mansfield SD. *Populus trichocarpa* cell wall chemistry and ultrastructure trait variation, genetic control and genetic correlations. *New Phytologist* 2013, 197:777-790.
3. Van Ooijen JW. JoinMap^®^4, Software for the calculation of genetic linkage maps in experimental populations. Kyazma B.V. 2006, Wageningen, Netherlands.
4. Tuskan, GA, et al. The genome of black cottonwood, *Populus trichocarpa* (Torr. & Gray). *Science* 2006, 313: 1596-1604.
5. Geraldes A, DiFazio S, Slavov GT, Priya R, **Muchero W** et al. A 34K SNP genotyping array for Populus trichocarpa: Design, application to the study of natural populations and transferability to other *Populus* species. ***Molecular Ecology Resources* 2013, 13:306-323.**
6. Li H. Durbin R. Fast and accurate short read alignment with Burrows-Wheeler transform. *Bioinformatics* 2009, 25: 1754–1760.
7. Li H, Handsaker B, Wysoker A, Fennell T, Ruan J et al. The Sequence Alignment/Map format and SAMtools. *Bioinformatics* 2009, 25: 2078–2079.
8. Citovsky V, Lee LY, Vyas S, Glick E, Chen MH, et al. Subcellular localization of interacting proteins by bimolecular fluorescence complementation in planta. *J Mol Biol* 2006, 362:1120-1131.
9. Guo J, Morrell-Falvey JL, Labbe JL, Muchero W, Kalluri UC, et al. Highly efficient isolation of *Populus* mesophyll protoplasts and its application in transient expression assays. *PLoS ONE* 2012, 7:e44908.
10. Gaillard C, Strauss F. Ethanol Precipitation of DNA with linear polyacrylamide as carrier. *Nucleic Acids Research* 1990, 18:378-378.
11. Ye J, Coulouris G, Zaretskaya I, Cutcutache I, Rozen S, et al. Primer-BLAST: A tool to design target-specific primers for polymerase chain reaction. *BMC Bioinformatics* 2012, 13:134.
12. [Zhong R](https://webmail.ornl.gov/owa/redir.aspx?C=_Zg3mLTEqEabhaStJLIhmkthO_SYc9BIIEGpwXyB97Gmrlv0NHmv0BGBEVjxeUV5xxFa2KCkwBo.&URL=http%3a%2f%2fwww.ncbi.nlm.nih.gov%2fpubmed%3fterm%3dZhong%2520R%255BAuthor%255D%26cauthor%3dtrue%26cauthor_uid%3d19965968), [Lee C](https://webmail.ornl.gov/owa/redir.aspx?C=_Zg3mLTEqEabhaStJLIhmkthO_SYc9BIIEGpwXyB97Gmrlv0NHmv0BGBEVjxeUV5xxFa2KCkwBo.&URL=http%3a%2f%2fwww.ncbi.nlm.nih.gov%2fpubmed%3fterm%3dLee%2520C%255BAuthor%255D%26cauthor%3dtrue%26cauthor_uid%3d19965968), [Ye ZH](https://webmail.ornl.gov/owa/redir.aspx?C=_Zg3mLTEqEabhaStJLIhmkthO_SYc9BIIEGpwXyB97Gmrlv0NHmv0BGBEVjxeUV5xxFa2KCkwBo.&URL=http%3a%2f%2fwww.ncbi.nlm.nih.gov%2fpubmed%3fterm%3dYe%2520ZH%255BAuthor%255D%26cauthor%3dtrue%26cauthor_uid%3d19965968). Functional characterization of poplar wood-associated NAC domain transcription factors. *[Plant Physiol](https://webmail.ornl.gov/owa/redir.aspx?C=_Zg3mLTEqEabhaStJLIhmkthO_SYc9BIIEGpwXyB97Gmrlv0NHmv0BGBEVjxeUV5xxFa2KCkwBo.&URL=http%3a%2f%2fwww.ncbi.nlm.nih.gov%2fpubmed%2f19965968)* 2010, 152: 1044-1055.
13. Zhong R, Ye ZH. The poplar Ptrwnds are transcriptional activators of secondary cell wall biosynthesis. *Plant Signal Behav* 2010, 5: 469-72.
14. Zhong R, McCarthy RL, Lee C, Ye ZH. Dissection of the transcriptional program regulating secondary wall biosynthesis during wood formation in poplar. *Plant Physiol* 2011, 157: 1452-68.
15. Norris SR, Meyer SE, Callis J. The Intron of *Arabidopsis-thaliana* polyubiquitin genes is conserved in location and is a quantitative determinant of chimeric gene-expression. *Plant Molecular Biology* 1993, 21:895-906.
