# Supplementary material for: High-resolution genetic mapping of allelic variants associated with cell wall chemistry in Populus
Source: BMC Genomics. 2015 Jan 23;16(1):24. doi: 10.1186/s12864-015-1215-z (PMC4307895; doi:10.1186/s12864-015-1215-z)
Supplement: Additional file 3: — QTL mapping results. [file 12864_2015_1215_MOESM3_ESM.docx]

Additional file 3. QTL intervals identified based on Multiple QTL-mapping Model (MQM) analysis in an inter-specific pseudo-backcross population.

| Trait | QTL physical interval | QTL interval size (Mb) | SNP marker at peak | LOD score | LOD significance threshold | % phenotypic variance explained |
| --- | --- | --- | --- | --- | --- | --- |
| S/G ratio_2nd year | 2665310-3243550 | 0.578 | scaffold_14_2979511 | 5.72 | 2.00 | 5.0 |
| S/G ratio_2nd year | 6573660-7137886 | 0.564 | scaffold_14_7068969 | 8.14 | 2.00 | 7.0 |
| S/G ratio_2nd year | 7229597-8291972 | 1.060 | scaffold_14_7559196 | 8.73 | 2.00 | 7.5 |
| S/G ratio_2nd year | 9862197-10623454 | 0.761 | scaffold_14_10087714 | 4.91 | 2.00 | 4.3 |
| S/G ratio_2nd year | 10681281-11239975 | 0.559 | scaffold_14_10904219 | 4.12 | 2.00 | 3.6 |
|  |  |  |  |  |  |  |
| S/G ratio_3rd year | 2665310-3632536 | 0.967 | scaffold_14_2979511 | 5.92 | 2.00 | 5.2 |
| S/G ratio_3rd year | 6375294-7137886 | 0.762 | scaffold_14_7068969 | 8.58 | 2.00 | 7.4 |
| S/G ratio_3rd year | 7176568-8291972 | 1.115 | scaffold_14_7559196 | 8.32 | 2.00 | 7.2 |
| S/G ratio_3rd year | 9840268-10623454 | 0.783 | scaffold_14_10122944 | 4.84 | 2.00 | 4.3 |
| S/G ratio_3rd year | 10661488-11331689 | 0.670 | scaffold_14_10904219 | 4.03 | 2.00 | 3.6 |
|  |  |  |  |  |  |  |
| Percent lignin_2nd year | 2182986-2849504 | 0.666 | scaffold_14_2388217 | 4.36 | 2.10 | 3.8 |
| Percent lignin_2nd year | 4195712-5481428 | 1.286 | scaffold_14_5327320 | 5.25 | 2.10 | 4.6 |
| Percent lignin_2nd year | 5718542-6375294 | 0.657 | scaffold_14_6007026 | 6.82 | 2.10 | 5.9 |
| Percent lignin_2nd year | 7559196-8291972 | 0.732 | scaffold_14_7697860 | 7.02 | 2.10 | 6.1 |
| Percent lignin_2nd year | 10209778-11160856 | 0.951 | scaffold_14_10681281 | 4.40 | 2.10 | 3.9 |
|  |  |  |  |  |  |  |
| Percent lignin_3rd year | 2182986-2849504 | 0.666 | scaffold_14_2388217 | 3.84 | 2.10 | 3.4 |
| Percent lignin_3rd year | 3715413-5481428 | 1.766 | scaffold_14_5327320 | 4.53 | 2.10 | 4.0 |
| Percent lignin_3rd year | 5530072-6449632 | 0.920 | scaffold_14_6043560 | 6.00 | 2.10 | 5.2 |
| Percent lignin_3rd year | 7347161-8291972 | 0.945 | scaffold_14_7697860 | 5.80 | 2.10 | 5.1 |
| Percent lignin_3rd year | 9862197-11397489 | 1.535 | scaffold_14_10864383 | 4.27 | 2.10 | 3.8 |
|  |  |  |  |  |  |  |
| 5-carbon sugars_2nd year | 4089011-5481428 | 1.392 | scaffold_14_5327320 | 2.74 | 2.00 | 2.4 |
| 5-carbon sugars_2nd year | 5530072-6243964 | 0.714 | scaffold_14_6007026 | 3.13 | 2.00 | 2.8 |
| 5-carbon sugars_2nd year | 7176568-8414841 | 1.238 | scaffold_14_7697860 | 2.55 | 2.00 | 2.3 |
|  |  |  |  |  |  |  |
| 6-carbon sugars_2nd year | 2182986-2849504 | 0.667 | scaffold_14_2594879 | 2.15 | 2.00 | 1.9 |
| 6-carbon sugars_2nd year | 5530072-6375294 | 0.845 | scaffold_14_6007026 | 3.61 | 2.00 | 3.2 |
| 6-carbon sugars_2nd year | 7229597-8717549 | 1.488 | scaffold_14_7697860 | 3.52 | 2.00 | 3.1 |
