# Supplementary material for: High-resolution genetic mapping of allelic variants associated with cell wall chemistry in Populus
Source: BMC Genomics. 2015 Jan 23;16(1):24. doi: 10.1186/s12864-015-1215-z (PMC4307895; doi:10.1186/s12864-015-1215-z)
Supplement: Additional file 4: — 34 K Infinium array-based association mapping results. [file 12864_2015_1215_MOESM4_ESM.docx]

Additional file 4. SNP-trait associations based on Mixed Linear Model (MLM) association mapping using genotypic data from the *Populus* 34K Infinium array.

| Trait_environment | SNP marker | Significance ranking (out of 1,439 SNPs) | *p-*value | *R^2^* | df | Nearest candidate gene |
| --- | --- | --- | --- | --- | --- | --- |
|  |  |  |  |  |  |  |
| pyMBMS *m/z* 135_Clatskanie | scaffold_14_2980220 | 1^st^ | 1.27E^-05†^ | 0.044 | 522 | Potri.014G036500 (Amino acid transporter) |
| Arabinose_Surrey | scaffold_14_2977633 | 1^st^ | 5.63E^-04‡^ | 0.029 | 334 |  |
|  |  |  |  |  |  |  |
| Glucose/xylose release_Native | scaffold_14_3027959 | 1^st^ | 6.57E^-06†^ | 0.040 | 678 | Potri.014G037200 (Kanadi transcription factor) |
| Glucose/xylose release_Native | scaffold_14_3028120 | 1^st^ | 6.57E^-06†^ | 0.040 | 678 |  |
| Glucose release_Native | scaffold_14_3027959 | 1^st^ | 1.38E^-05†^ | 0.038 | 678 |  |
| Glucose release_Native | scaffold_14_3028120 | 1^st^ | 1.38E^-05†^ | 0.038 | 678 |  |
| Xylose release_Native | scaffold_14_3027959 | 2^nd^ | 6.62E^-04‡^ | 0.025 | 678 |  |
| Xylose release_Native | scaffold_14_3028120 | 2^nd^ | 6.62E^-04‡^ | 0.025 | 678 |  |
| pyMBMS *m/z* 125_Clatskanie | scaffold_14_3028570 | 12th | 8.55E^-04‡^ | 0.028 | 523 |  |
|  |  |  |  |  |  |  |
| Xylose release_Corvallis | scaffold_14_7043301 | 1^st^ | 1.06E^-05†^ | 0.090 | 260 | Potri.014G089400 (Angustifolia CtBP transcription factor) |
| Glucose/xylose release_Native | scaffold_14_7044284 | 3^rd^ | 6.84E^-04‡^ | 0.020 | 679 |  |
|  |  |  |  |  |  |  |
| pyMBMS *m/z* 120_Clatskanie | scaffold_14_7055338 | 1^st^ | 1.32E^-05†^ | 0.044 | 523 | Potri.014G089700 (Copper transport protein ATOX1-related) |
| Average wood density_Surrey | scaffold_14_7053760 | 1^st^ | 9.18E^-05‡^ | 0.036 | 334 |  |
| Average wood density_Surrey | scaffold_14_7053739 | 2^nd^ | 9.75E^-05‡^ | 0.036 | 334 |  |
| Percent lignin_Clatskanie | scaffold_14_7054863 | 2^nd^ | 2.72E^-04‡^ | 0.028 | 594 |  |
| Percent lignin_Clatskanie | scaffold_14_7054809 | 3^rd^ | 2.73E^-04‡^ | 0.028 | 592 |  |
|  |  |  |  |  |  |  |
| 5-carbon sugars_Native | scaffold_14_7969314 | 1^st^ | 1.55E^-06†^ | 0.048 | 600 | Potri.014G101900 (Ca^2+^ transporting ATPase) |
| 6-carbon sugars_Native | scaffold_14_7969314 | 1^st^ | 5.00E^-05‡^ | 0.037 | 600 |  |
| pyMBMS *m/z* 102_Clatskanie | scaffold_14_7971054 | 2^nd^ | 8.31E^-05‡^ | 0.036 | 524 |  |
|  |  |  |  |  |  |  |
| pyMBMS *m/z* 235_Clatskanie | scaffold_14_10865898 | 1^st^ | 3.00E^-05†^ | 0.040 | 520 | Potri.014G142700 (Protein kinase) |
| S and G monomers_ Surrey | scaffold_14_10865898 | 1^st^ | 7.99E^-05‡^ | 0.038 | 330 |  |
| Percent S monomers_Surrey | scaffold_14_10865898 | 1^st^ | 9.49E^-05‡^ | 0.037 | 330 |  |
| Glucose release_Native | scaffold_14_10867394 | 3^rd^ | 5.59E^-04‡^ | 0.026 | 585 |  |

^†^ Significant at the chromosome-wise Bonferroni adjusted *p*-value

^‡^ Significant at the QTL-interval-wise Bonferroni adjusted *p*-value, SNPs within ± 1 LOD value of the QTL peak were considered
