# Supplementary material for: High-resolution genetic mapping of allelic variants associated with cell wall chemistry in Populus
Source: BMC Genomics. 2015 Jan 23;16(1):24. doi: 10.1186/s12864-015-1215-z (PMC4307895; doi:10.1186/s12864-015-1215-z)
Supplement: Additional file 5: — Whole-genome resequencing-based association mapping results. [file 12864_2015_1215_MOESM5_ESM.docx]

| Trait_environment | SNP marker | *p*-value | Significance threshold | df | *R^2^* | Nearest candidate gene |
| --- | --- | --- | --- | --- | --- | --- |
| 6-carbon sugars_Corvallis | scaffold_14_2983445 | 1.14E^-07^ | 6.85E^-05^ | 223 | 0.21 | Potri.014G036500 (Amino acid transporter) |
| 5-carbon sugars_Corvallis | scaffold_14_2983445 | 1.49E^-06^ | 6.85E-^05^ | 223 | 0.18 |  |
| Glucose/xylose release_Corvallis | scaffold_14_2969670 | 3.86E^-06^ | 6.85E^-05^ | 223 | 0.13 |  |
| Glucose/xylose release_Corvallis | scaffold_14_2968007 | 4.69E^-06^ | 6.85E^-05^ | 223 | 0.12 |  |
| Glucose release_Clatskanie | scaffold_14_2986800 | 6.54E^-05^ | 6.85E^-05^ | 381 | 0.06 |  |
| Glucose release_Clatskanie | scaffold_14_2986816 | 6.54E^-05^ | 6.85E^-05^ | 381 | 0.06 |  |
| Glucose release_Clatskanie | scaffold_14_2986033 | 6.83E^-05^ | 6.85E^-05^ | 381 | 0.06 |  |
| pyMBMS *m/z* 135_Clatskanie | scaffold_14_2976865 | 4.17E^-06^ | 6.85E^-05^ | 382 | 0.07 |  |
| pyMBMS *m/z* 135_Clatskanie | scaffold_14_2980220 | 1.19E^-05^ | 6.85E^-05^ | 382 | 0.06 |  |
| pyMBMS *m/z* 135_Clatskanie | scaffold_14_2979305 | 1.39E^-05^ | 6.85E^-05^ | 382 | 0.06 |  |
| pyMBMS *m/z* 135_Clatskanie | scaffold_14_2980895 | 4.48E^-05^ | 6.85E^-05^ | 382 | 0.05 |  |
| pyMBMS *m/z* 135_Clatskanie | scaffold_14_2982151 | 4.48E^-05^ | 6.85E^-05^ | 382 | 0.05 |  |
|  |  |  |  |  |  |  |
| Glucose release_Clatskanie | scaffold_14_3025294 | 8.89E^-05^ | 1.13E^-04^ | 381 | 0.07 | Potri.014G037200 (Kanadi transcription factor) |
| Glucose release_Corvallis | scaffold_14_3035343 | 8.64E^-05^ | 1.13E^-04^ | 223 | 0.06 |  |
|  |  |  |  |  |  |  |
| 6-carbon sugars_Clatskanie | scaffold_14_7053121 | 1.96E^-05^ | 8.36E^-05^ | 382 | 0.06 | Potri.014G089700 (Copper transport protein ATOX1-related) |
| Percent lignin_Clatskanie | Scaffold_14_7050421 | 5.09E^-05^ | 8.36E^-05^ | 382 | 0.05 |  |
|  |  |  |  |  |  |  |
| Xylose release_Corvallis | scaffold_14_7970015 | 9.03E^-06^ | 1.02E^-04^ | 223 | 0.11 | Potri.014G101900 (Ca^2+^ transporting ATPase) |
| 5-carbon sugars_Native | scaffold_14_7966546 | 5.61E^-05^ | 1.02E^-04^ | 428 | 0.04 |  |
| 5-carbon sugars_Native | scaffold_14_7972112 | 6.18E^-05^ | 1.02E^-04^ | 428 | 0.04 |  |
| 5-carbon sugars_Native | scaffold_14_7960908 | 9.17E^-05^ | 1.02E^-04^ | 428 | 0.04 |  |
|  |  |  |  |  |  |  |
| Xylose release_Corvallis | scaffold_14_10855744 | 5.49E^-07^ | 5.10E^-05^ | 223 | 0.20 | Potri.014G142700 (Protein kinase) |
| Glucose/xylose release_Corvallis | scaffold_14_10855744 | 4.80E^-06^ | 5.10E^-05^ | 223 | 0.13 |  |
| Glucose release_Corvallis | scaffold_14_10860927 | 6.49E^-06^ | 5.10E^-05^ | 223 | 0.13 |  |
| Glucose/xylose release_Corvallis | scaffold_14_10860927 | 1.56E^-05^ | 5.10E^-05^ | 223 | 0.12 |  |
| 6-carbon sugars_Corvallis | scaffold_14_10891547 | 9.36E^-06^ | 5.10E^-05^ | 223 | 0.15 |  |
| 5-carbon sugars_Corvallis | scaffold_14_10891547 | 2.69E^-05^ | 5.10E^-05^ | 223 | 0.13 |  |

Additional file 5. SNP-trait associations based on Mixed Linear Model (MLM) association mapping using whole genome re-sequencing genotype data for the QTL intervals on scaffold 14.
