# Supplementary material for: High-resolution genetic mapping of allelic variants associated with cell wall chemistry in Populus
Source: BMC Genomics. 2015 Jan 23;16(1):24. doi: 10.1186/s12864-015-1215-z (PMC4307895; doi:10.1186/s12864-015-1215-z)
Supplement: Additional file 6: — Protein alignment showing allelic variants of the Angustifolia CtBP transcription factor Potri.014G089400. [file 12864_2015_1215_MOESM6_ESM.docx]

Potri.014G089400_BESC-293 MSATTTRSLATMSHRRNTNTPPPPQQQQQQQQQQQQQQQRLPLVVTLNCI 50

Potri.014G089400_BESC-470 MSATTTRSLATMSHRRNTNTPPPPQQQQQQQQQQQQ--QRLPLVVTLNCI 48

************************************ ************

Potri.014G089400_BESC-293 EDFAIEQDSLSGVALIEHVPLGRLSDGKIESAAAVLLHSLAYLPRAAQRR 100

Potri.014G089400_BESC-470 EDFAIEQDSLSGVALIEHVPLGRLSDGKIESAAAVLLHSLAYLPRAAQRR 98

**************************************************

Potri.014G089400_BESC-293 LRPYQLILCLGSADRAVDSALAADLGLRLVHVDTSRAEEIADTVMALFLG 150

Potri.014G089400_BESC-470 LRPYQLILCLGSADRAVDSALAADLGLRLVHVDTSRAEEIADTVMALFLG 148

**************************************************

Potri.014G089400_BESC-293 LLRRTHLLSRHALSASGWLGSLQPLCRGMRRCRGLVLGIVGRSASARSLA 200

Potri.014G089400_BESC-470 LLRRTHLLSRHALSASGWLGSLQPLCRGMRRCRGLVLGIVGRSASARSLA 198

**************************************************

Potri.014G089400_BESC-293 TRSLAFKMSVLYFDVHEGPGKLTRSSITFPLAARRMDTLNDLLAASDLIS 250

Potri.014G089400_BESC-470 TRSLAFKMSVLYFDVHEGPGKLTRSSITFPLAARRMDTLNDLLAASDLIS 248

**************************************************

Potri.014G089400_BESC-293 LHCALTNETVQIINEECLQHIKPGAFLVNTGSSQLLDDCALKQLLIDGTL 300

Potri.014G089400_BESC-470 LHCALTNETVQIINEECLQHIKPGAFLVNTGSSQLLDDCALKQLLIDGTL 298

**************************************************

Potri.014G089400_BESC-293 AGCALDGAEGPQWMEAWVKEMPNVLILPRSADYSEEVWMEIREKAISILQ 350

Potri.014G089400_BESC-470 AGCALDGAEGPQWMEAWVKEMPNVLILPRSADYSEEVWMEIREKAISILQ 348

**************************************************

Potri.014G089400_BESC-293 SFFFDGIVPKNAVSDEEGEESEIGDESEQFHRQDKESTLQDSVGEQLTDD 400

Potri.014G089400_BESC-470 SFFFDGIVPKNAVSDEEGEESEIGDESEQFHRQDKESTLQDSVGEQLTDD 398

**************************************************

Potri.014G089400_BESC-293 IQLTPETSRKKVSGQSIESTSQAQGSGMSQNTTTRSDERRSRSGKKAKKR 450

Potri.014G089400_BESC-470 IQLTPETSRKKVSGQSIESTSQAQGSGMSQNTTTRSDERRSRSGKKAKKR 448

**************************************************

Potri.014G089400_BESC-293 HGRQKPRQKSDNPSQLEKESTSHQEDDTAMSGSDQVSSSRFASPEDSRSR 500

Potri.014G089400_BESC-470 HGRQKPRQKSDNPSQLEKESTSHQEDDTAMSGSDQVSSSRFASPEDSRSR 498

**************************************************

Potri.014G089400_BESC-293 KTPIELMQESSSGQLSRSGKRLSGKSDELLKDGHIIALYARDRPALHVSR 550

Potri.014G089400_BESC-470 KTPIELMQESSSGQLSRSGKRLSGKSDELLKDGHIIALYARDRPALHVSR 548

**************************************************

Potri.014G089400_BESC-293 QRAKGGGWFLDALSNVTKRDPAAQFLVVFRNKDTIGLRSFAAGGKLLQIN 600

Potri.014G089400_BESC-470 QRAKGGGWFLDALSNVTKRDPAAQFLVVFRNKDTIGLRSFAAGGKLLQIN 598

**************************************************

Potri.014G089400_BESC-293 RRMEFVFTSHSFDVWESWMLEGSLDECRLVNCRNPLAILDARVEILA**T**IA 650

Potri.014G089400_BESC-470 RRMEFVFTSHSFDVWESWMLEGSLDECRLVNCRNPLAILDARVEILA**A**IA 648

*********************************************** **

Potri.014G089400_BESC-293 EDDGVTRWLD 660

Potri.014G089400_BESC-470 EDDGVTRWLD 658

**********
