# Supplementary figures and images for: High-resolution genetic mapping of allelic variants associated with cell wall chemistry in Populus
Source: BMC Genomics. 2015 Jan 23;16(1):24. doi: 10.1186/s12864-015-1215-z (PMC4307895; doi:10.1186/s12864-015-1215-z)

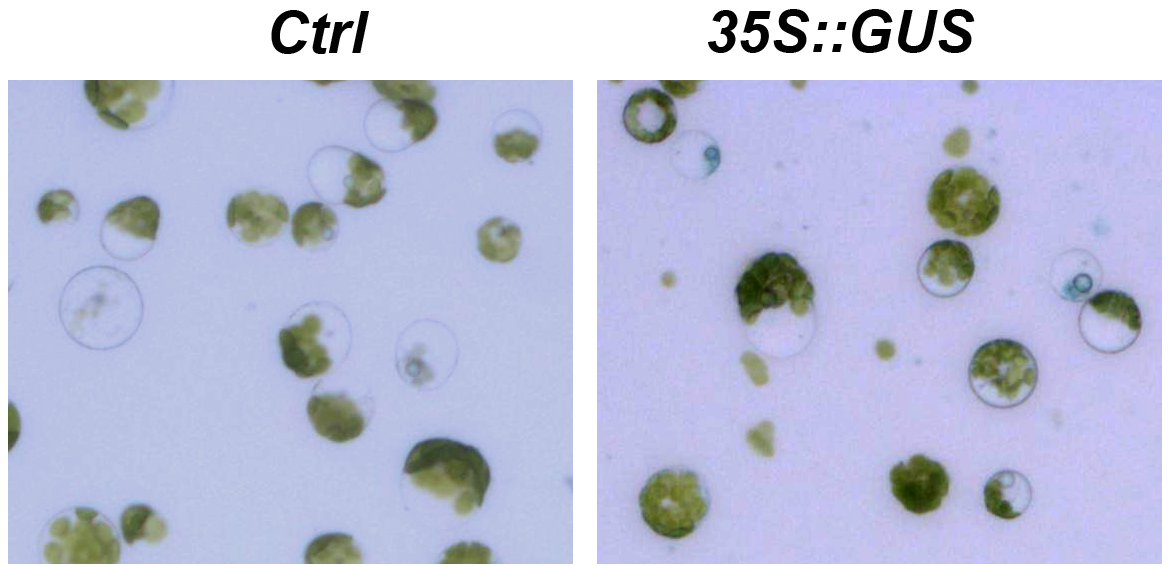

Supplement: Additional file 7: — Validation of the constructed transient over-expression vector using the GUS reporter assay. [file 12864_2015_1215_MOESM7_ESM.tiff]

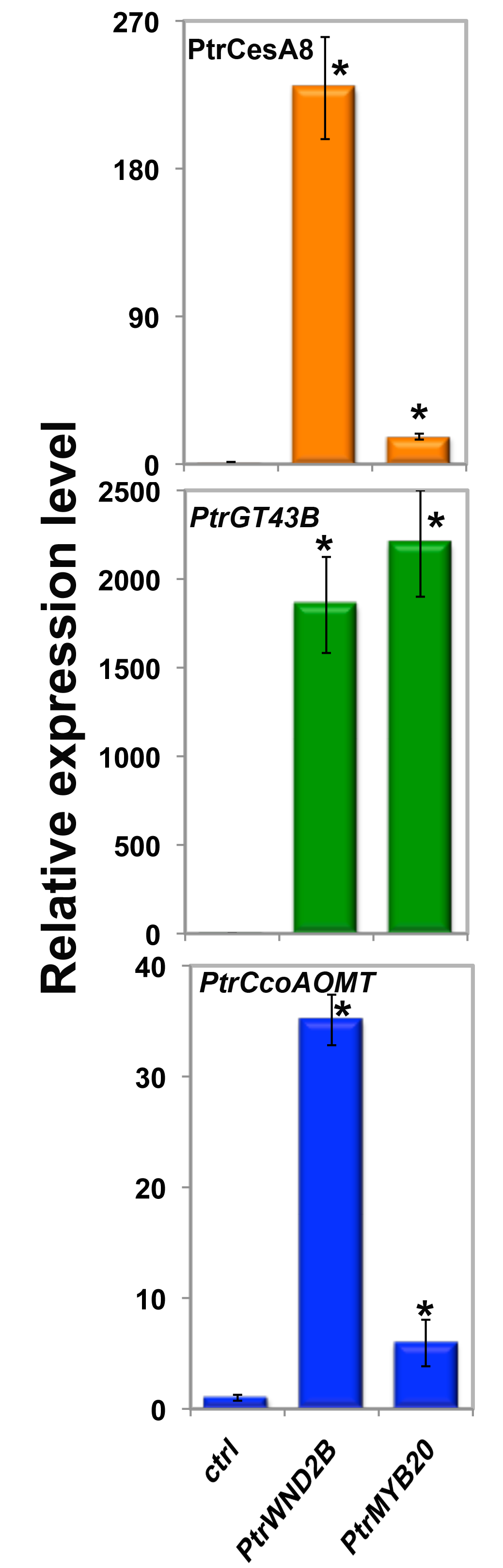

Supplement: Additional file 8: — Validation of the feasibility of using Populus protoplasts as a tool to study regulation of reporter genes for the cellulose, hemicellulose and lignin biosynthesis pathways. [file 12864_2015_1215_MOESM8_ESM.tiff]
