# Supplementary material for: High-resolution genetic mapping of allelic variants associated with cell wall chemistry in Populus
Source: BMC Genomics. 2015 Jan 23;16(1):24. doi: 10.1186/s12864-015-1215-z (PMC4307895; doi:10.1186/s12864-015-1215-z)
Supplement: Additional file 10: — Phenotypic variation in mapping populations. [file 12864_2015_1215_MOESM10_ESM.docx]

Additional File 10. Minimum, mean and maximum phenotypic values for cell-wall recalcitrance traits segregating in a *P. trichocarpa* x *P. deltoides* pseudo-backcross pedigree and a population of diverse *P. trichocarpa* genotypes.

| Phenotype_environment | Min | Mean | Max | *n* |
| --- | --- | --- | --- | --- |
|  |  |  |  |  |
| Percent lignin_Pedigree_2008 | 21.8 | 26.9 | 30.7 | 515 |
| Percent lignin_Pedigree_2010 | 23.2 | 26.4 | 32.7 | 514 |
| S/G ratio_Pedigree_2008 | 1.5 | 2.0 | 2.5 | 515 |
| S/G ratio_Pedigree_2010 | 1.5 | 2.0 | 2.5 | 514 |
| 5-carbon sugars_Pedigree_2008 | 23.7 | 29.2 | 34.4 | 515 |
| 6-carbon sugars_Pedigree_2008 | 24.8 | 29.9 | 36.7 | 515 |
| Percent lignin_Native | 15.7 | 22.7 | 27.9 | 797 |
| Percent lignin_Corvallis | 20.6 | 25.7 | 28.0 | 300 |
| Percent lignin_Clatskanie | 18.4 | 24.5 | 28.1 | 926 |
| S/G ratio_Native | 1.0 | 2.1 | 3.0 | 797 |
| S/G ratio_Corvallis | 1.5 | 1.9 | 2.4 | 300 |
| S/G ratio_Clatskanie | 1.3 | 1.9 | 2.5 | 926 |
| Glucose release_Native (mg/mg biomass) | 0.01 | 0.30 | 0.48 | 786 |
| Glucose release_Corvallis (mg/mg biomass) | 0.01 | 0.12 | 0.21 | 300 |
| Glucose release_Clatskanie (mg/mg biomass) | 0.17 | 0.37 | 0.50 | 926 |
| Xylose release_Native (mg/mg biomass) | 0.07 | 0.13 | 0.19 | 786 |
| Xylose release_Corvallis (mg/mg biomass) | 0.01 | 0.11 | 0.19 | 300 |
| Xylose release_Clatskanie (mg/mg biomass) | 0.09 | 0.19 | 0.24 | 926 |
| Glucose/xylose release_Native (mg/mg biomass) | 0.17 | 0.43 | 0.69 | 786 |
| Glucose/xylose release_Corvallis (mg/mg biomass) | 0.02 | 0.22 | 0.33 | 300 |
| Glucose/xylose release_Clatskanie (mg/mg biomass) | 0.27 | 0.55 | 0.66 | 926 |
| 5-carbon sugars_Native | 18.1 | 24.5 | 29.9 | 797 |
| 5-carbon sugars_Corvallis | 19.5 | 23.3 | 31.7 | 300 |
| 6-carbon sugars_Native | 21.8 | 31.0 | 43.2 | 797 |
| 6-carbon sugars_Corvallis | 20.3 | 25.4 | 38.3 | 300 |
